# Ultrahigh-Throughput Enzyme Engineering and Discovery in *In Vitro* Compartments

**DOI:** 10.1021/acs.chemrev.2c00910

**Published:** 2023-05-01

**Authors:** Maximilian Gantz, Stefanie Neun, Elliot J. Medcalf, Liisa D. van Vliet, Florian Hollfelder

**Affiliations:** Department of Biochemistry, University of Cambridge, 80 Tennis Court Rd, Cambridge CB2 1GA, U.K.

## Abstract

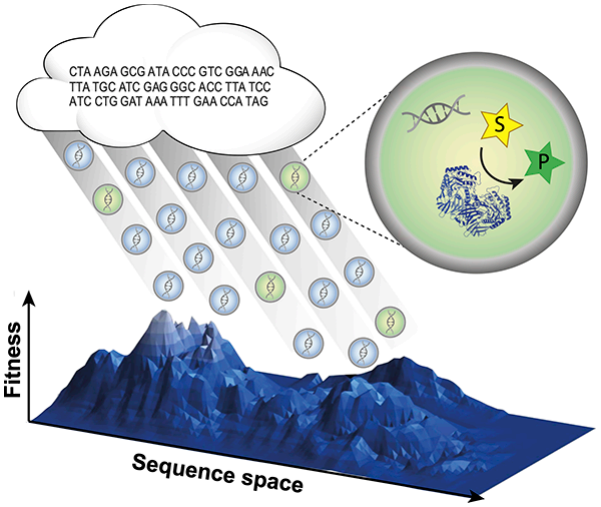

Novel and improved biocatalysts are increasingly sourced from libraries via experimental screening. The success of such campaigns is crucially dependent on the number of candidates tested. Water-in-oil emulsion droplets can replace the classical test tube, to provide *in vitro* compartments as an alternative screening format, containing genotype and phenotype and enabling a readout of function. The scale-down to micrometer droplet diameters and picoliter volumes brings about a >10^7^-fold volume reduction compared to 96-well-plate screening. Droplets made in automated microfluidic devices can be integrated into modular workflows to set up multistep screening protocols involving various detection modes to sort >10^7^ variants a day with kHz frequencies. The repertoire of assays available for droplet screening covers all seven enzyme commission (EC) number classes, setting the stage for widespread use of droplet microfluidics in everyday biochemical experiments. We review the practicalities of adapting droplet screening for enzyme discovery and for detailed kinetic characterization. These new ways of working will not just accelerate discovery experiments currently limited by screening capacity but profoundly change the paradigms we can probe. By interfacing the results of ultrahigh-throughput droplet screening with next-generation sequencing and deep learning, strategies for directed evolution can be implemented, examined, and evaluated.

## 1 Introduction

**Figure 1:**
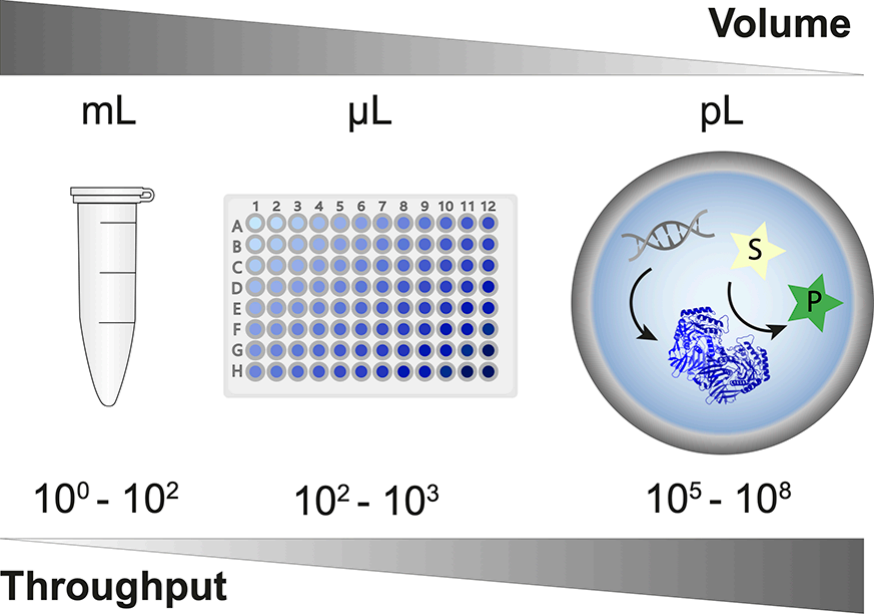
Droplet microfluidics enables a massive scale-down of reaction volumes from milliliters in test tubes, beyond microliters used in plate formats (and robotic liquid handling systems) to picoliters in *in vitro* compartments. This miniaturization format is highly economical, so access to ultrahigh-throughput screening of enzymes (here shown as generated by *in vitro* expression, but see Figure 8 8 for other formats) becomes possible at relatively low cost. This review provides an overview of the use of droplet compartmentalization in protein discovery and engineering.

Protein engineering by directed evolution relies on combinatorial experiments that explore how amino acids are best arranged to bring about functional molecules. New functional proteins are in high demand in applications ranging from affinity reagents or antibodies in medical research and therapy to biocatalysts for “green”, energy efficient and sustainable processes. Finding these molecules is difficult because the total combinatorial diversity generated from 19 amino acid alternatives in every position of a protein is enormous and efficient methods for its exploration are required to find catalysts on a useful time scale. To increase the chances of success and to accelerate library screening, the throughput should be as high as possible (Figure 1).

Water-in-oil emulsion droplets, made and handled in microfluidic devices, provide a relatively recently established experimental format for screening and selection of functional proteins. The droplet compartment replaces the classical test tube (or multiwell plate), and lab-on-a-chip devices automatize and miniaturize liquid handling operations—carried out by one’s own fair hands or by large robots—so that experiments can be conducted more quickly, with minimal consumption of reagents and plasticware (tubes, plates, and tips). The micrometer dimension of droplet compartments achieves a scale-down of reaction volumes to the picoliter range (corresponding to a >10^7^-fold volume reduction compared to the regular 96-well plate format with a ∼200 μL volume).^1,2^ This is necessary because the possible combinations of amino acids - even in a focused protein library - easily exceed the screening capacity (e.g., a library in which only 5 residues are fully randomized almost matches the throughput of droplet microfluidics; 20^5^ = 3.2 × 10^6^ combinations).

For screening of protein libraries in directed evolution or functional metagenomics, each droplet compartment needs to contain a code for the identity of the library member: the droplet boundary thus links genotype and phenotype by compartmentalizing the gene, enzyme, and reaction product. The criterion for selecting individual variants is a readout of the successful progress of the reaction of interest (ideally directly reporting quantitatively on product concentration), so an analytical interface is necessary to evaluate the reaction progress.

In the future, protein engineering campaigns may go beyond the “black box” lottery that combinatorial screening experiments currently are: one can never be sure whether a library contains initial hits that can be evolved later—and why. When next-generation sequencing will be applied to the output of rounds of screening, one will produce large data sets that describe ensembles of genes satisfying an experimentally set threshold. These correlations of sequence to function could help to describe “fitness landscapes”. When trajectories through sequence space are visualized, directed evolution ceases to be a “black box”. Instead “fitness landscape” maps may help to steer directed evolution by evaluating whether navigation into more or less interesting sections of sequence space is possible. Ideally long trajectories familiar from natural evolution should be emulated in laboratory experiments. Machine-learning algorithms and artificial intelligence^3−5^ will be helpful to obtain insight into multiparameter spaces and in all likelihood be necessary to provide meaningful extrapolations from experimentally explored sequences to further improved proteins.

A large number of excellent reviews describe technical aspects of *in vitro* compartmentalization and droplet microfluidics, along with various applications.^6−19^ The objective of this review is to take stock of the steps that have been established as the basis for the discovery of functional enzymes in large libraries, to showcase studies that have integrated droplet technologies with protein discovery campaigns, to provide a guide for newcomers into this area faced with everyday issues of practical implementation, and finally to extrapolate where this technology will find its most powerful uses.

## 2 Types of *In Vitro* Compartments

**Figure 2:**
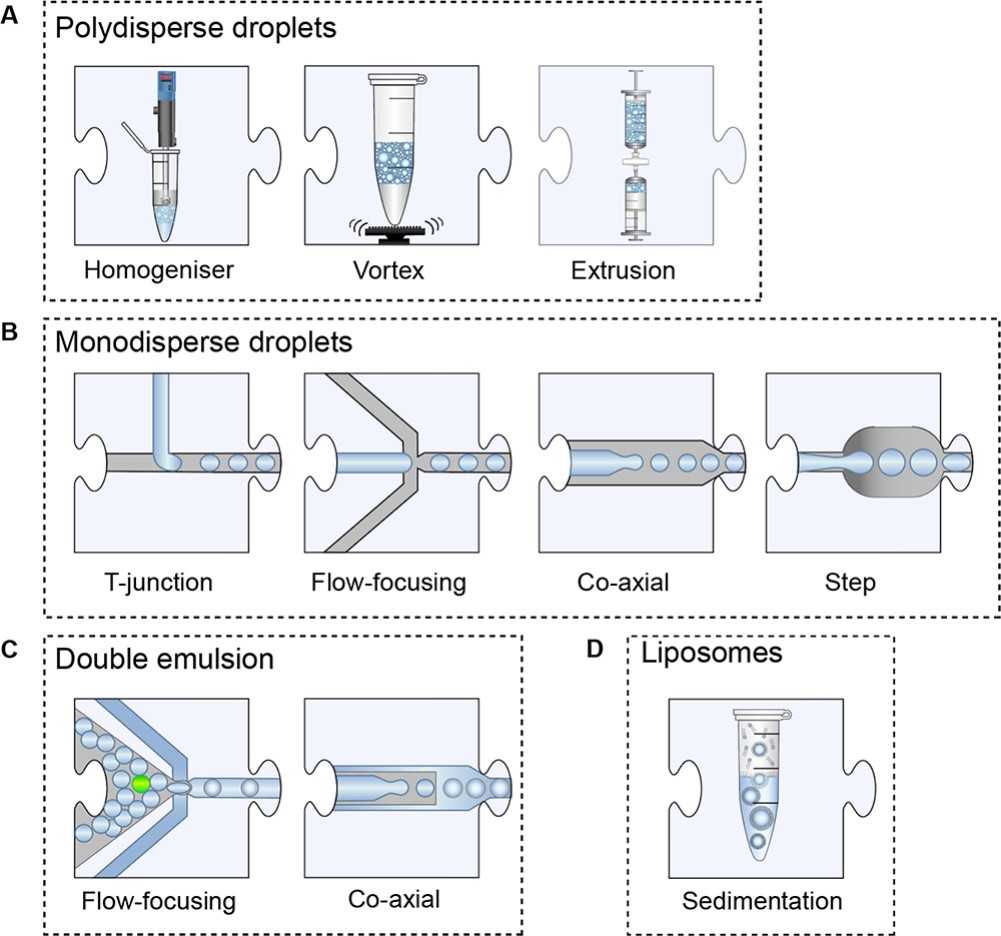
Droplet generation units. (A) Polydisperse water-in-oil droplets are generated via a homogenizer, simply by vortexing or by extruding an emulsion across a filter. For the production of double-emulsion droplets, the process is repeated with the first emulsion in an aqueous carrier phase. (B) Monodisperse droplets are generated in microfluidic devices of varying designs: (1) a T-Junction, (2) a flow-focusing junction, (3) the coaxial flow of the two fluids, or (4) a step device. (C) Double emulsions are generated by flowing water-in-oil droplets into an aqueous carrier phase by using the same geometries that are used for generating monodispersed droplets. (D) Liposomes are generated by the sedimentation of an emulsion through a lipid monolayer and into a second aqueous phase.

Conceptually the idea of isolating a single library member from all others by a droplet boundary is embodied by a large number of formats (Table 1). These *in vitro* compartments differ in size, ease of production, stability, and the rate at which they can be generated. Historically, water-in-oil (W/O) emulsion droplets were first produced in a *polydisperse* format (for single-cell^20,21^ and single-enzyme^22^ experiments), where droplets are generated very quickly. However, while the droplet boundary restricts crosstalk, droplet sizes vary considerably and the assay quality may be less than uniform, as differently sized droplets will contain different amounts of reagents. Nevertheless, polydisperse emulsions still are used today for protein engineering.^23−28^ 10^10^–10^11^ compartments are produced in minutes: (i) with a stirring bar,^29,30^ (ii) with an emulsifier or homogenizer,^18,27,31^ (iii) by vortexing,^32,33^ or by extrusion through a filter^26,34^ (Figure 2A).

**Table 1:**
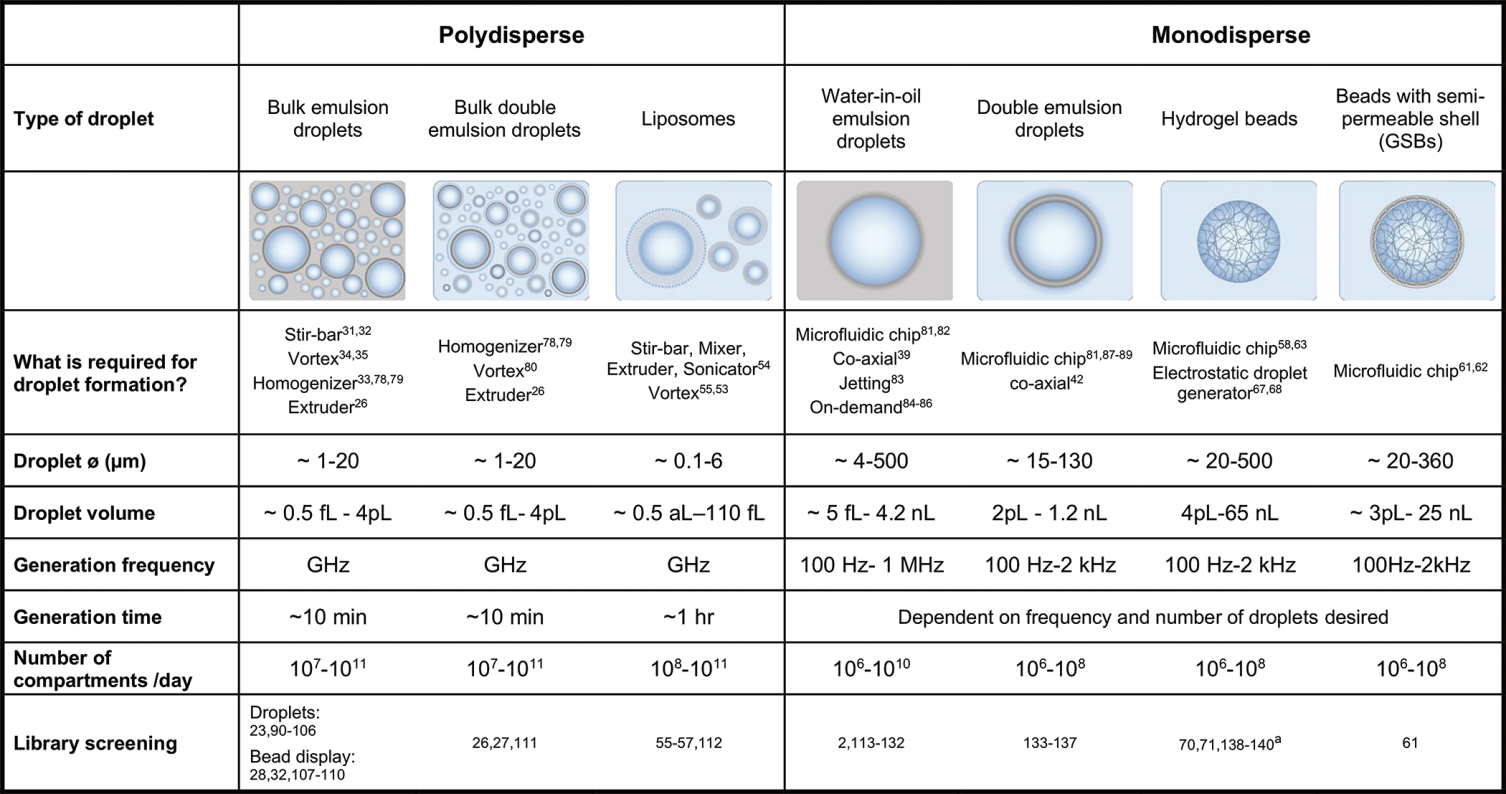
Polydisperse and Monodisperse Droplet Compartments Used for Protein Engineering

a Self-encapsulation of cells by hydrogel formation: “fur-shell”.

The ease of setup makes polydisperse formats attractive, but the difference in droplet size within one experiment may often preclude screening based on relatively small activity differences. On the other hand, a larger number of droplets can be generated in an instant using the polydisperse format. Especially for reactions in which the product is amplified (as in polymerase selections^23−25^), rendering them quasi-binary yes/no selections, polydisperse emulsions are particularly suitable.^35^ Nevertheless, quantitative screenings for reactions that generate an optically active product are also possible,^27^ and an even subdivision of a screening output into bins has been successful, despite some noise in the sequencing readout.^28^

The microfluidic production of *monodisperse* emulsions allows a more stringent quantification of the reaction product based on the optical readout.^500,501,80^ There is also an additional level of control in microfluidics: multistep workflows can be constructed; the timing of lysis, reaction, and incubation, and other steps can be precisely governed. The production of monodisperse water-in-oil emulsions^36^ is not instantaneous, even if it occurs at kHz frequencies, with a output of >10^8^ compartments (with diameters of a few μm) per day. A large number of microfluidic device designs that achieve near-ideal monodispersity (0.2 to 3% coefficient of variation of the droplet radius)^37−42^ are available (Figure 2B), e.g., flow-focusing devices,^36^ T-junctions,^43−45^ coaxial/capillary,^37,46,47^ or step^48−50^ designs.

Monodisperse as well as polydisperse droplets can be emulsified once again to produce water-in-oil-in-water (W/O/W) “double emulsions” that overall have rheological and electrostatic properties of an aqueous solution, which means that they can be analyzed in widely used commercial devices that are optimized e.g. for cell sorting in flow cytometers (see below).

Liposome compartments can be generated by vortexing a mixture of amphiphilic lipids (e.g., phospholipids such as phosphocholines (POPCs), phospho-glycerol (POPG), phospho-serine (POPS) or a cholesterol mixture) with an aqueous phase to generate a W/O emulsion, which is placed on top of the final outer solution followed by centrifugation (Figure 2D).^51^ Alternatively, stirring followed by extrusion and sonication^52^ can bring about vesicle compartments. Despite being generally less stable than emulsions, vesicles can be sorted by fluorescence-activated cell sorters (FACS). This method of “liposome display” has been used to evolve membrane proteins that benefit from being anchored in the hydrophobic ring around the vesicle^53,54^ as well as an aminoacyl-tRNA synthetase.^55^

**Figure 3:**
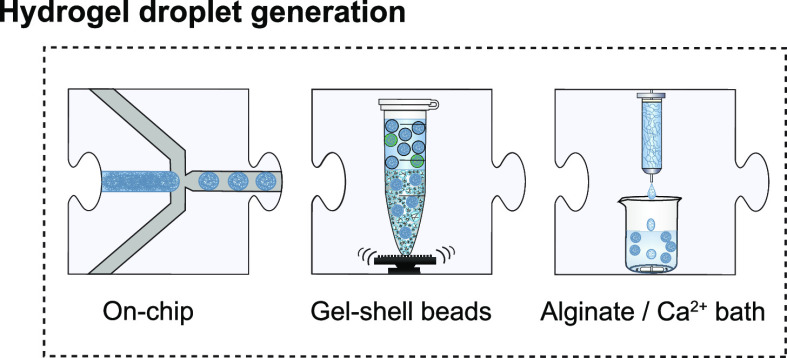
Nanoliter hydrogel bead generation. Hydrogels can be used as the aqueous phase for water-in-oil droplet generation on a chip employing the various generation designs (Figure 2). When agarose and alginate droplets are de-emulsified into a positively charged polymeric solution, a layer-by-layer semipermeable shell is formed around the hydrogel. Similarly, the laminar-jet breakup of an alginate solution into a calcium bath generates monodisperse hydrogel beads.

An alternative to liquid compartments is to turn the droplet into a microsphere made of a soft material: gel-shell beads (GSBs) “immortalize” the compartmentalization by generating an agarose microsphere with a selectively permissible boundary from a droplet. After encapsulation of all reaction components in monodisperse droplets together with additional components (agarose and alginate), the droplet contents solidify to form a gel upon lowering the temperature,^56−58^ and thus bead microspheres (Ø ∼ 25 μm) are generated. Subsequent to the removal of the droplet boundary, the deposition of layers of polyelectrolytes on the surface of these microspheres (based on charge interactions between negative alginate in the gel and positive polyammonium electrolyte) creates a size-selective shell (with permeability only for molecules < 2 kDa). Thus, reaction products (when tagged e.g. to an oligonucleotide) can be captured together with enzyme and its encoding plasmid DNA, creating a genotype–phenotype linkage. Such GSBs have been sorted by FACS in a directed evolution campaign.^59^ Hollow-core polyelectrolyte-coated chitosan alginate microcapsules (HC-PCAMs) have been similarly endowed with selective permeability and used to demonstrate enrichment of a sortase (employing a large particle sorter (COPAS, complex object parametric analyzer and sorter) instead of FACS).^60^ Alternative materials provide routes to producing hydrogel beads as microspheres: alginate can be solidified with cations on-chip (Figure 3)^61−64^ or by laminar jetting into a bath,^65−69^ and polyacrylamide can be cross-linked.^70,71^ Beads based on hydrogels and other materials (e.g., polystyrene or paramagnetic composites) can also be used as a template to generate near-monodisperse droplets that tightly wrap around the bead via vortexing^72−74^ or pipetting through filter tips^28,75^ into an oil phase, avoiding microfluidic devices altogether.

## 3 Modular Workflows and Their Operation

In conventional laboratory work, our hands (or liquid handling robots) carry out the basic tasks that an experiment entails. For scaled-down experiments in microdroplets, samples have to be processed in an entirely different way. In the last decades, a number of chip designs have emerged from the “lab-on-a-chip” community that provide a repertoire of “units of manipulation”. Workflow design would “translate” each manipulation carried out manually in a large-scale experiment (e.g., adding or removing reagents by hand, carrying out an optical measurement as the basis for a sorting decision) into its on-chip equivalent and combine multiple unit operations into a sequence of steps. This modularity can be conveniently represented as jigsaw pieces. For example, Figure 2 shows multiple designs for ten alternatives for the first step of a microfluidic workflow, droplet formation (and three more for the formation of hydrogels can be found in Figure 3).

**Figure 4:**
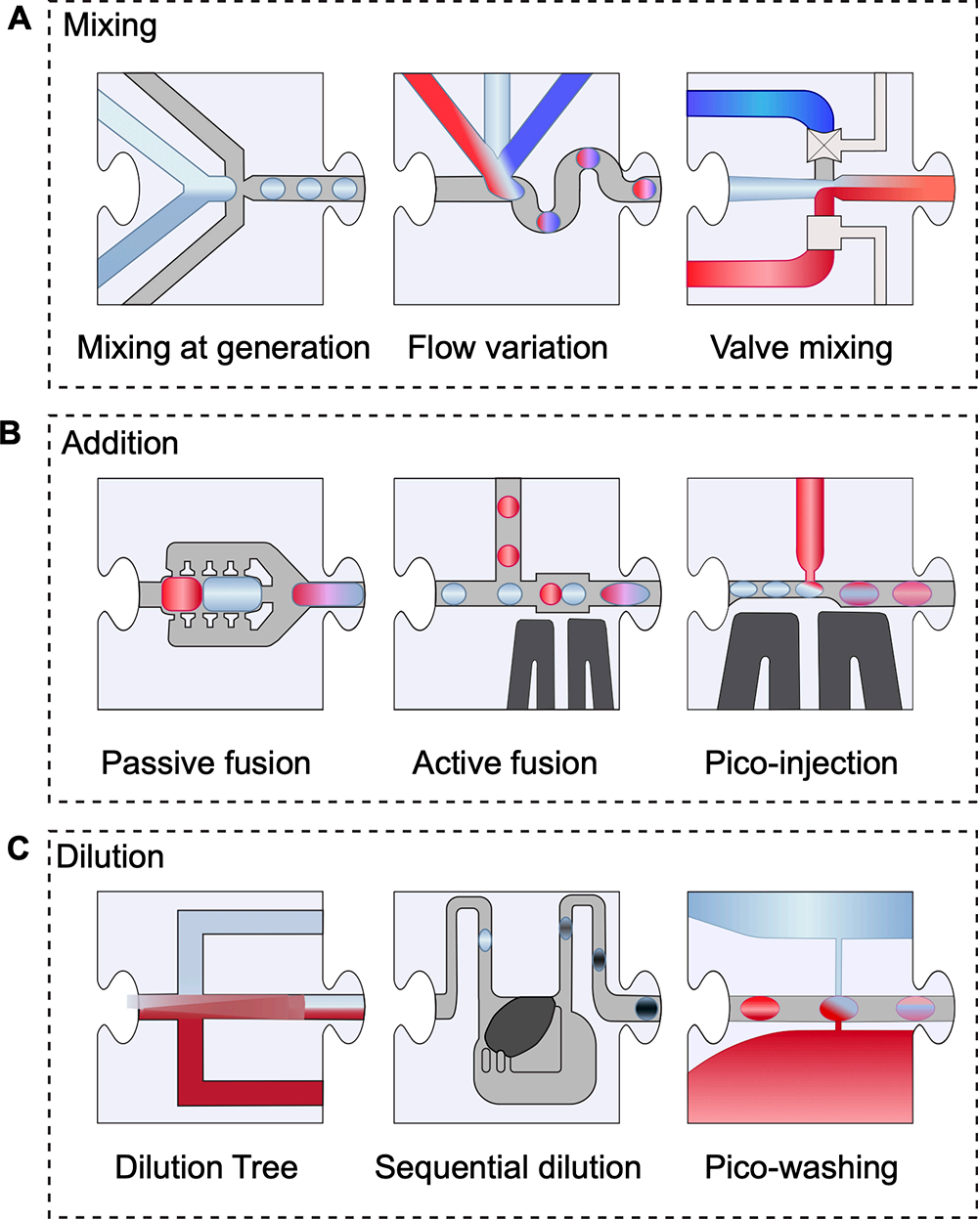
Droplet manipulation for mixing, adding, or diluting reagents. (A) Mixing of reagents can be accomplished on chip at the time of generation or by addition of reagents to droplets at a later stage. (B) Merging or fusion of droplets can be done either passively using various device designs or by electrocoalescence. (C) Dilution of the droplet content can be done directly on chip by varying the flow rates of the mixed aqueous phases during generation, controlling the flow and mixing via valve systems, separating a laminar flow in a tree-like design, fusing varying proportions of droplet pairs, simultaneously adding and removing reagents or generating droplets from sequentially diluting a concentrated initial reagent.

**Figure 5:**
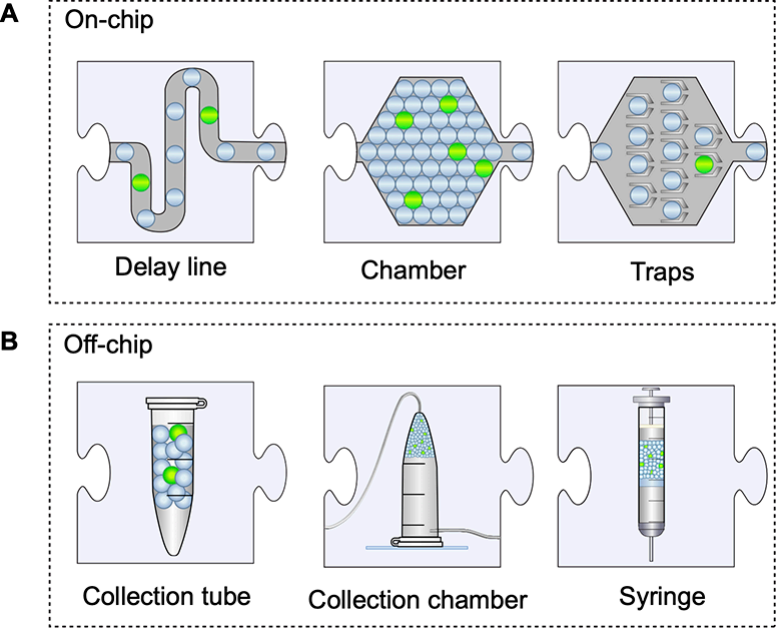
Droplet incubation. (A) Droplets can be incubated on a chip within a channel, packed in a chamber, or held in position by trapping features. (B) Droplets can be collected into any collection tube, or in chambers or directly into syringes for easy re-injection into other microfluidic devices for further manipulation, analysis, or sorting.

The workflow designer would pick one droplet formation module and combine it with the next unit of operation that replaces pipetting in classical experimentation: (i) mixing of reagents occurs by chaotic advection at the point of droplet formation,^44,139^ (ii) addition of reagents is achieved by droplet merging in passive fashion,^140^ by electrocoalescence of two droplets,^141−144^ or by picoinjection of an aqueous stream,^1,121,145,146^ and (iii) dilution of reagents (Figure 4). A recent addition to the toolkit is the “picowasher”, which enables simultaneous addition and subtraction of fluid from droplets, allowing washing of the droplet contents with or without solid particles inside.^147^ Once a biochemical reaction is set up with all of its components, the experimenter typically has to allow time for the reaction to proceed, and there are multiple on-chip solutions for this incubation step (Figure 5). Delay-lines keep the droplets in a predefined order (e.g., allowing time tracking of the incubation period), either in device microchannels,^117,148,149^ in long tubing,^122^ or in a capillary^150^ that connects two devices. Incubation times in the region of up to an hour are possible.^117,128,149^

For longer incubation times, the channels become so long that back-pressure typically builds up and challenges device stability (e.g., stability of droplet generation or delamination of the PDMS from the glass support). When delay-line incubation becomes impractical, incubation chambers or traps provide an on-chip opportunity to store droplets, albeit at the price of losing the rank order of the droplets. Such cavities can contain millions of droplets, and their size can be expanded when support pillars are included in the design.^151−153^ Droplets can also be hydrodynamically captured into traps^501,154−157^ or sink wells^49,158^ for longer-term analysis of droplet contents. While the order is still not easily controlled, time courses for individual droplets can be recorded as the basis for precise characterization of the reaction occurring in a sample of droplets.

Often it is more straightforward to carry out incubations offline instead: in standard Eppendorf tubes, in custom-built collection chambers,^121,131,159^ or in syringes^2,87^ up to 10^8^ droplets can be stored. After incubation, droplets are reinjected into a chip to be presented for sorting (see Section 5) or any other downstream modules. Re-injection is optimal when the droplets are tightly packed upon entry into the device because diluted droplets lead to an unequal spacing between droplets. Subsequent sorting devices operate with higher quality when the droplets are uniformly spaced.

When directed evolution for higher enzyme activity is successful, the timeframes in one experimental campaign will change: obviously depending on the intrinsic activity of an enzyme, but in addition also when the enzyme becomes faster from one selection round to the next. In such a case the chip design will have to be adjusted to raise the bar for selection by making the conditions more stringent. For example, Schnettler et al.^117^ started with an off-chip incubation/re-injection workflow but in subsequent stages of evolution, ended up with an integrated device. Here droplet generation, incubation, and sorting were combined, to take account of the ∼360-fold improvements that reduced the reaction times from 2–3 h to less than one hour. It is tempting to think that ultimately there will be one “directed evolution machine”, but the shifting timescales in directed evolution experiments make it necessary to customize workflows to accommodate the stage of proficiency and set the selection threshold according to the evolutionary strategy chosen. Rapid prototyping of chip devices is, therefore, necessary to accommodate enzymes with different activity levels and to keep up with evolutionary improvements, may they be large or small.

## 4 Chip Devices

**Figure 6:**
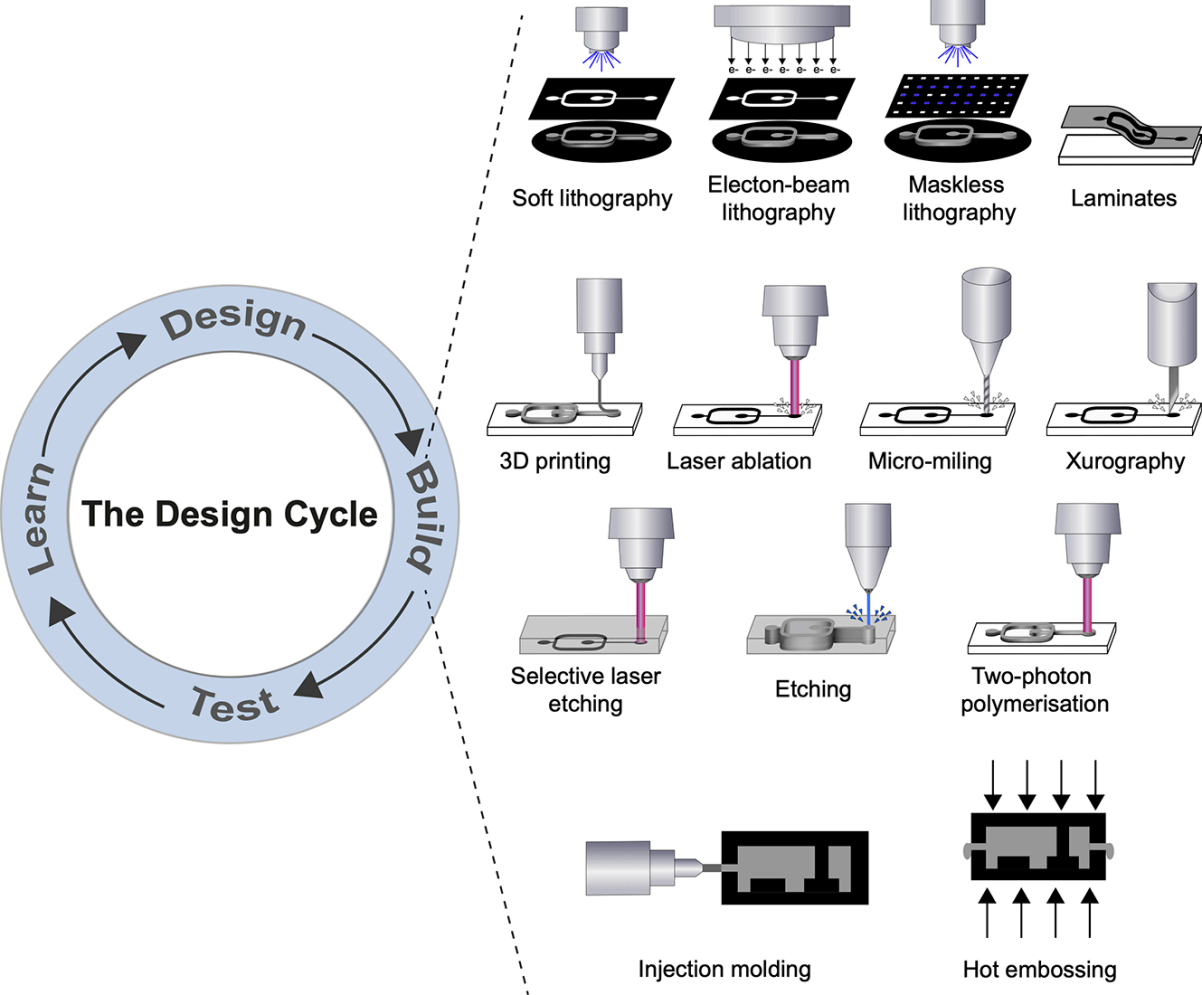
The design cycle for microfluidic chip devices and the main types of available current and future fabrication methods. Rapid design and redesign of prototypes that translate workflows from the macro- to the microscale on chips are necessary to establish new assays for a wider circle of reactions but also within one directed evolution campaign to adjust the design to the increasing proficiency of the evolved catalyst (that requires modified timings or expression, incubation, and/or different selection thresholds). *Soft lithography*: the most commonly used method; a photomask patterns the UV curing of a photoresist resin. *Electron-beam lithography*: relies on the deposition energy of the accelerated electrons to the resist film on the substrate using a photomask. *Maskless lithography*: similar to soft lithography; however, dynamic micron-sized apertures (e.g., DMDs, LCoS) replace a photomask to project the UV onto the photoresist resin. *Laminates*: several sheets of material are bonded together to form a total device, such as an interface layer, a flow layer, and a bottom layer. *3D printing*: an additive manufacturing technique whereby devices are formed from polymerized layers. *Laser ablation*: a laser removes material through vaporization; typically it is pulsed to reduce surface damage (e.g., cracking). *Micromilling*: uses an endmill (typically in the hundreds of microns) to drill away material in order to form channels. *Xurography*: uses a knife plotter to cut patterns out of thin films. *Selective laser etching*: a laser creates a pattern inside a glass-like material, which is then removed using an etchant. *Etching*: removes material from the surface using an etchant to create a pattern. *Injection molding*: prepolymerized pellets of a thermoplastic are heated and injected under pressure into a mold cavity and then cooled to solidify the material. *Two-photon polymerization*: a high-resolution technique whereby a localized area polymerizes at the focus of laser beam. *Hot embossing*: similar to injection molding, a thermoplastic is heated up in a mold and the pressure of two plates compresses the polymer into the desired shape.

Devices for generation capable of the key modular processes can be made by soft lithography in polydimethylsiloxane (PDMS) using standard protocols for rapid prototyping, i.e., iterative testing of designs in cycles (Figure 6) that take a few days, followed by an experimental test (and redesign in response to failures). The soft lithography process is split into two steps: creating the master mold and forming the polymeric device. To create the “master”, several lithographic techniques involve the deposition of a thin layer of SU-8 photoresist onto a silicon wafer by spin-coating and “soft baking”. Ultraviolet light is then passed through a photomask (glass or plastic etc.) to pattern the photoresist that is subsequently “post baked”. The unpolymerized photoresist is dissolved using propylene glycol monomethyl ether acetate (PGMEA).^160^ Finally, the wafer can then be coated with a fluorinated silane to adjust channel hydrophobicity.^161^ In the second step, PDMS is poured into the master mold, baked to form the polymerized device, bonded onto glass (or another PDMS surface) via oxygen plasma treatment, and coated with fluorinated silane for hydrophobicity.^161,162^ The silanization of the PDMS devices serves to reduce “wetting effects” or friction at the channel walls,^163^ and various surface modifications for hydrophobic or hydrophilic coating are available to match the carrier phase, allowing choices of different oils.^15^

So-called “2.5D” designs (i.e., varying channel depth within the device) can be created by patterning several layers on the master in an iterative process. In this way, areas of the mask can have an additional buildup of material, leading to varying channel depths within the device. The channel system can be connected to pumps and reservoirs via tubing that is inserted into holes made with biopsy punches. Such devices are perfectly suitable for directed evolution campaigns, although delamination and the soft nature of the material mean that the devices have a limited lifetime.

Many other harder materials (e.g., glass, poly(methyl methacrylate) (PMMA)) can be used analogously, and devices can be bought “off-the-shelf” from several companies (e.g., microfluidic ChipShop, Dolomite, and Darwin Microfluidics). Briefly, the choice of device material depends on the application. Inorganic materials (e.g., glass) are durable and rigid, making them very reusable but also more difficult and costly to fabricate. Elastomers (e.g., PDMS) are flexible and can be fabricated more rapidly through soft lithography, but they suffer from delamination issues at high pressures. Thermoplastics are easier to scale-up in production (using hot embossing and injection molding) but become more difficult to manufacture at a smaller scale due to the need for expensive micromachining tools (for an extensive review see ref (15)).

Microfluidic designs are generated with AutoCAD, Fusion360, or other computer-aided design software, and the resultant designs are converted into a mask for soft lithographic fabrication (or an STL file for 3D printing). The open access availability of AutoCAD templates (e.g., deposited in DropBase,^164^ Grabcad,^165^ or Metafluidics^166^) makes previously tested designs accessible. It should be noted that *ab initio* design and complex fluid modeling are not prerequisites for working chips. Rapid prototyping of PDMS devices facilitates design–build–test–learn cycles within a few days that are often equally instructive (and readily accessible even for neophytes). Figure 6 summarizes alternative prototyping methods used by companies and in academic settings, and Table 2 profiles their scopes.

**Table 2:**
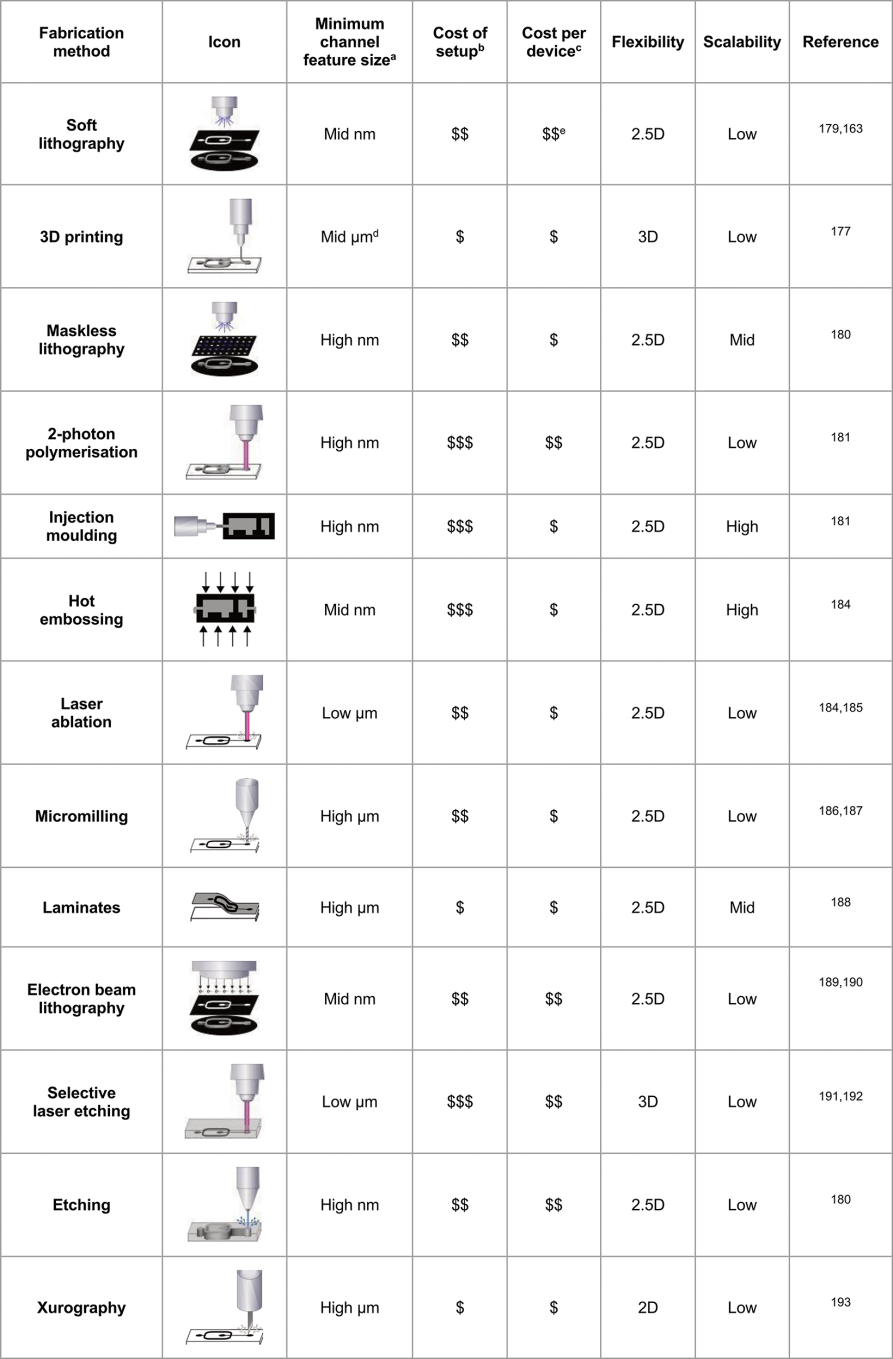
Benchmarks for Common Microfluidic Fabrication Technologies That Provide Criteria for Choosing Which Method Suits the Desired Features and Costs of a Chip Device

a Low: 1–10; mid: 10–100; high: 100–1000.

b Cost of setup: “$”: $1000s; “$$”: $10,000s; “$$$”: $100,000s.

c Cost per device: “$”: $1–10; “$$”: $10–100; “$$$”: >$100.

d Microprojection lithography is much smaller but also more costly and time-intensive.

e This is the cost of fabricating a new master mold; replicating a design from the mold is much less expensive. Ranges for ^b^ and ^c^ are the authors’ best estimates.

The device design depends on turnover rates: fast reactions require integrated modules on a chip,^128^ while slower reactions invite discontinuous processes with off-chip storage for incubation. However, the experimental time scales of different enzyme reactions (and mutants with increasing activities in one experiment) mean that device designs must be frequently adjusted. Soft lithography remains an option for these iterations, but alternative chip manufacturing technologies may soon replace this method. Table 2 gives a breakdown of the advantages and disadvantages of different fabrication techniques. 3D printing has seen a rise in popularity due to the decreasing costs of 3D printers, a decrease in minimum feature size, and the ability to create true 3D channels,^167−172^ and it would conveniently automate chip manufacture. A race is on for miniaturizing the channel features to match the μm resolution of the masks used for making PDMS chips. Fused deposition modeling (FDM) 3D printing involves injection of a heated, liquified polymer through a nozzle onto an XYZ stage to “paint” a device design (i.e., build up a three-dimensional structure layer-by-layer).^173^ Here the minimal channel dimensions have been shown to be just 58 × 65 μm.^174^ SLA/DLP (stereolithography/digital light processing) or projection micro-stereolithography 3D printing builds up material through the polymerization of a photopolymer using a guided laser beam or a configurable mask.^175^ When light is guided or projected through a mask to a photopolymer (which then is cured), features are created to achieve flow channel cross sections down to 18 × 20 μm.^176^ The benefits of 3D printing are the flexibility of the materials used, increased fabrication speed, ease of use, and ability to rapidly share designs globally.^173^ However, more development is required to develop inexpensive systems that produce smaller channels.

The lab around the chip is crucial for the operation of a microfluidic device. A standard instrumental setup includes a pump (syringe or pressure pumps), an inverted microscope, a high-speed camera, a computer with control software, syringes, and tubing. Pressure is provided to the syringes through the action of pumps, generally using syringe pumps or vacuum pumps. Due to the high speeds that are used in droplet microfluidics, typically, droplets flow in the kHz range, and a high-speed camera is needed to look at the functioning and routing of the droplets in a human-accessible time scale. Computer control is provided as proprietary software (e.g., for pump operation) or is custom-built using several programs such as LabView or custom-written software (e.g., Python-based). Concerted efforts to share software would be highly beneficial for the user community, helping to avoid reinventing the wheel and making an interdisciplinary research area easier to navigate for newcomers. Sharing software or code is possible via OpenWetWare or GitHub (see e.g. our repositories^164,177^).

## 5 Detection and Sorting

The optical transparency of the device material makes interrogation of droplet contents possible when an optical probe is integrated into the biological assay carried out in a droplet (Table 3). An optical signal reporting on the concentration of reaction product is then translated into a sorting decision.

**Table 3:**
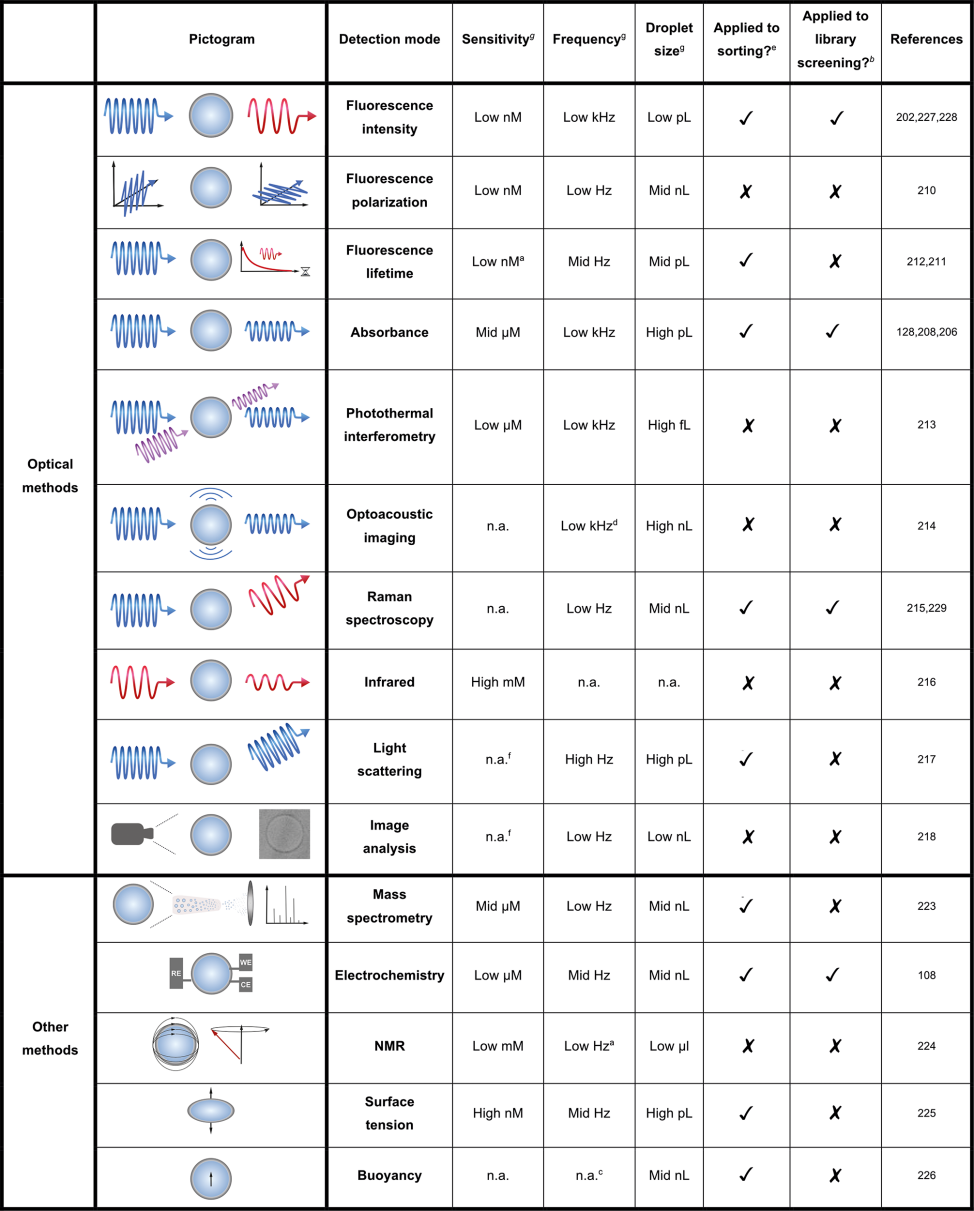
Overview of Detection Modes Currently Available for Microfluidic Setups

a Estimated from graphs provided or related literature.

b Applied in a screening of enzyme activity from a functional metagenomic or directed evolution library.

c Passive selection: in theory the throughput is only limited by the droplet generation frequency.

d Only the B-scan rate is shown, not how quickly droplets can be measured.

e Referring to *any* sorting experiment, i.e. an enrichment experiment or a library screening (not necessarily of enzyme activity and not necessarily monoclonal).

f Used for cells, no molar detection limit available.

g Low, 1–10; mid, 10–100; high, 100–1000; n.a. not applicable or available.

Fluorogenic assays are the most sensitive: when fluorescein is a reaction product, as little as 3000 molecules can be detected per droplet (corresponding to a low nanomolar concentration in picoliter droplet volumes),^118^ based on laser-induced fluorescence. The small reaction volume means that the enzyme concentration can easily be higher than the detectable fluorescein product concentration: >40,000 copies of GFP can be generated from one template molecule by *in vitro* expression^151^ or >10^6^ copies of an enzyme from lysis of a single cell:^126^ this means that fewer than a single turnover per enzyme molecule is comfortably detectable. Paradoxically the extreme miniaturization in droplets thus increases sensitivity compared to plate-based screens. While finding a highly efficient enzyme is the ultimate goal of a discovery campaign, early stages of directed evolution or metagenomic screening often involve low-activity catalysts (with an initially weak, promiscuous activity as a springboard for improvements)^193−195^ that are inefficiently expressed in a heterologous host. For these targets, fluorescence provides access to crucial starting points for evolutionary campaigns.

In addition to practical shortcomings (e.g., photobleaching), limits of fluorescence detection emerge when precise fine-tuning of enzymes for substrates that do not have a fluorogenic group is required. Fluorescein is bulky and hydrophobic, so it is potentially very different in terms of molecular recognition from natural functional groups. As a leaving group it is much more reactive (p*K*_a_ 6.4) than natural leaving groups (e.g. sugars, p*K*_a_ 12–14). Often improvements for a fluorescein-containing model substrate translate into a concomitant increase in the activity of substrates that e.g. have a different leaving group.^117,196^ However, this improvement is typically smaller due to specialization for the fluorogenic substrate—following directed evolution’s basic law, “*you get what you screen for*”.^197^

**Figure 7:**
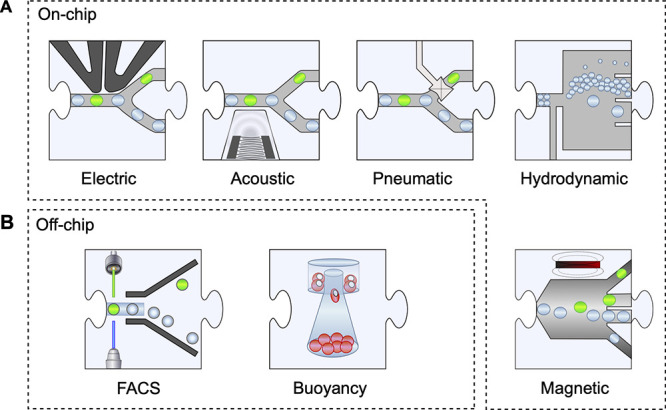
Sorting. Following analysis of the contents of individual droplets for product formation (using the methods listed in Table 3) sorting decisions are taken and droplets are steered into a collection bin for positive hits (whereas without intervention they would move into an outlet). In two cases of self-sorting, the content of the droplets causes the physical properties of the entire droplet to change, so that hydrodynamics or buoyancy becomes indicative of reaction progress.

Most cases of successful library selections on-chip (see Figure 7, Table 3) were based on coupling fluorescence detection with dielectrophoresis,^198−201^ in which an electrode (0.5–2 kV) is triggered by the optical signal (FADS, fluorescence-activated droplet sorting). kHz screening rates can be achieved (routinely with rates similar to a flow cytometer of 1–8 kHz,^117,118,123,127,128,196^ but even achieving up to 30 kHz^202^). Most screens are based on a single fluorophore, but selection based on multiple color detection has also been demonstrated.^203^ Other sorting methods are shown in Figure 7.

It is important to note that water-in-oil emulsions cannot be sorted in most flow cytometers (FACS, fluorescence-activated cell sorters) because the majority use an aqueous sheath fluid as a carrier phase and are incompatible with an oil phase carrying water-in-oil emulsion droplets. However, alternative droplet formats exist to replace the on-chip sorter with a FACS. Single emulsions are emulsified again to produce water-in-oil-in-water “double emulsions” that overall have rheological and electrostatic properties of an aqueous solution and are amenable to FACS (Figure 2C).^87^ The multistep processes described in the preceding section can still be carried out when the second emulsification step is performed last. Polydisperse single emulsions can be converted into double emulsions using a homogenizer,^27,76^ by vortexing,^78^ or by filter extrusion.^26^ When the monodispersity is to be retained, on-chip re-emulsification of monodisperse single emulsions is possible.^40,86,87^ Liposomes behave as double-emulsion droplets and can be sorted in FACS.^54,55^ Likewise, formats in which a bead is carrying genotype and phenotype can be sorted by FACS, which has been employed for the selection of protein binders,^75,204^ kinases,^28^ or triesterases.^30^

FACS and on-chip sorters operate with similar throughputs, >10^7^ per day, so both methods are similarly powerful. On-chip workflows allow setting up more complex processes (see below, Figure 9), but FACS sorting of double emulsions removes a technical complication and, with only a droplet-formation step performed on-chip, will be much easier to implement in nonspecialist laboratories. For widening the circle of users, a sorting step that only requires access to a walk-in instrument, e.g. in a centralized facility, will be highly attractive and help to popularize droplet approaches to a broader audience. However, FACS is limited to fluorogenic assays and serves only a relatively narrow range of target reactions. Also current multistep workflow protocols (see below, Figure 9) are only feasible while the droplets are on the chip, but when converted to double emulsions, microfluidic on-chip processing ceases to be an option.

Absorbance detection has more recently emerged as an alternative detection mode to enlarge the reactions of interest to chromogenic assays and can be coupled with dielectrophoretic sorting, named absorbance-activated droplet sorting (AADS) in analogy to the FADS described above. Practically, AADS is attractive: the setup is more straightforward and less expensive than FADS, as no lasers or photomultiplier tubes are needed. On the other hand, detection is not as sensitive as fluorescence detection (high μM vs nM detection limits, respectively). Absorbance is directly proportional to path length; therefore, droplets with a larger diameter (and therefore larger volumes) are needed. Consequently, the amount of reagent required for each droplet is larger, and the throughput of sorting is reduced because a higher electric field is needed to sort larger droplets.

In current enzyme screening campaigns, FADS was at least ∼20-fold faster than AADS (1–3 kHz^198,201^ vs 100 Hz).^126^ Attempts to increase the sensitivity and sensitivity of absorbance sorting have been made: (i) Duncombe et al.^205^ introduced UVADS (UV–Vis Spectra Activated Droplet Sorter) in a channel design with increased path length (by installing a right-angled turn at the detection interface) and by recording entire spectra (200–1050 nm) as unique signatures in UV–Vis Spectra-Activated Droplet Sorting. (ii) Richter et al.^206^ have shown kHz sorting throughput in a model separation based on removal of droplet trace artifacts by using a combination of surface acoustic waves and microlenses in the form of an optical air cavity. (iii) Medcalf et al.^207^ overcame the scattering caused by droplet edges in an improved microfluidic design (i.e., with a single-layered inlet leading to enabling more even spacing), refractive index matching, and faster sorting algorithms (compared to ref (126)), so sorting around 1 kHz became possible.

Fluorescence anisotropy (or fluorescence polarization) is a similarly sensitive detection technology to distinguish between bound and unbound forms of the fluorescently labeled analyte. Here, the fluorophore—attached away from the place of binding or catalysis—is excited using linearly polarized light, and the ratio between vertically and horizontally polarized emission light provides information about the rotational lifetime or tumbling of the fluorescently labeled substrate. This effectively provides a size measurement that has been used on droplets for assessment of binding processes.^208,209^ Extending this approach to catalysis (e.g., of size-changing protease or glycosidase reaction) will be useful to assay biopolymer-degrading or -assembling enzymes, but the integration into a sorter is necessary.

Fluorescence lifetime assays require a longer measuring time than the above-mentioned fluorescence assays (>ms instead of <μs), but in recent experiments fluorescence lifetime-activated droplet sorting (FLADS) has been shown to operate with frequencies in the 60–100 Hz range.^210,211^

Many other optical detection techniques have been developed: photothermal interferometry,^212^ optoacoustic imaging,^213^ Raman,^214^ infrared imaging,^215^ light scattering,^216^ and image analysis.^217^ While opening the option for different screening modalities, they all have reduced sensitivity, with the highest, photothermal interferometry, being at a low μM concentration. Methods to increase sensitivity and enable screening^214^ have been developed and successfully used in sorting a diacylglycerol acyltransferase library.^218^ The frequency of these techniques varies, with photothermal interferometry and optoacoustic imaging managing kHz speeds, while the others are at 1–100 Hz speeds.

A very attractive detection method is mass spectrometry (MS), because it is label-free, potentially possible with any ionizable product, and also provides information on multiple product candidates (and their ratios) emerging from an enzymatic reaction. Electrospray ionization has been used in several studies, after phase separation,^219^ directly from biphasic systems (double emulsions)^220^ or from plugs in segmented flow.^221^ In one case, an enzyme activity screening has been demonstrated: Holland-Moritz et al.^222^ enabled this by splitting droplets on-chip into two queues, one to be analyzed by ESI-MS and the other for dielectrophoretic sorting in response to the MS result (with addition of marker droplets for synchronization). Now sequences and functional readout could be matched, albeit with a throughput of <1 Hz. In these seminal experiments, >10^6^ copies of the DNA template had to be supplied in the droplets. Library selections would require droplets to be monoclonal (at least initially); therefore, integration with DNA amplification may be necessary. It will also need to be checked whether *in vitro* expression produces enough protein to yield detectable quantities of product if its ionization is difficult.

Other non-optical methods have been developed: electrochemistry^106^ and NMR.^223^ These methods both work at a much lower frequency (1–10 Hz) due to the need for a longer interrogation time and the need for a large droplet volume. Surface tension-^224^ and buoyancy-based^225^ detection have also been applied to droplet sorting, with potentially very high throughputs possible for buoyancy screening due to passive selection.

We envision more progress on label-free detection methods to be developed to match conventional microfluidic sorting speeds due to the obvious advantage of not needing a labeled substrate or product. This circumvents lengthy assay development times and prevents evolving enzymes that are not specific to the target of interest but to the label itself. However, sensitivity issues and the length of the interrogation time need further development.

Additionally, other sorting mechanisms are in exploration, e.g. (i) hydrodynamic “self-sorting” of differently sized droplets^229−231^ or of droplets with different buoyancy;^225^ (ii) magnetic sorting^232^ based on the encapsulation of magnetic particles that enable pulling droplets into a sorting channel; and (iii) sorting with pneumatic valves (via actuation of a valve that opens or closes a channel).^233−,235^

## 6 Expression Systems

The identity of library members is defined by a DNA identifier—a gene or a plasmid or fosmid in a cell—depending on whether an *in vitro* or *in vivo* expression is used to generate protein. The DNA is supplied at the start of an experiment into emulsion droplets in a Poisson distributed fashion. Here, Poisson’s equation describes the probabilistic likelihood of the occupation of a droplet compartment with 0, 1, or more. Ideally, droplets are monoclonal, i.e., initially containing just one library member, so a Poisson distribution, in which single compartmentalization dominates (while the majority of droplets is typically empty), is chosen, e.g., in directed evolution experiments.

**Figure 8:**
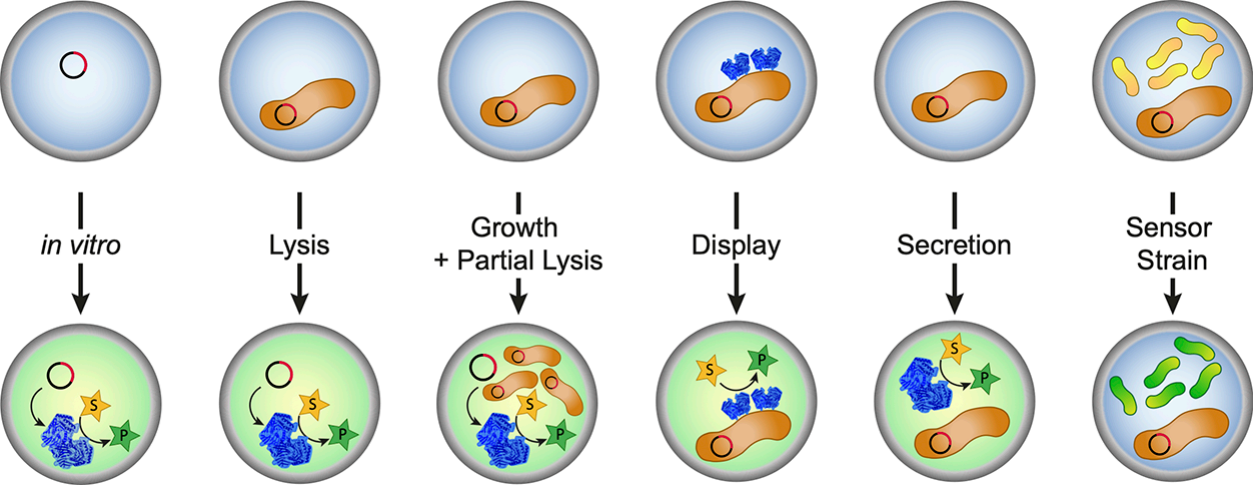
Expression systems used in droplets. Single library members are encapsulated in droplets according to Poisson distributions where they encounter the reaction substrate. For *in vitro* systems DNA library members are compartmentalized and expressed using cell-free expression systems. Alternatively, cells representing library members (and containing the genotype) are compartmentalized: while the encounter with substrate is straightforward for display systems (e.g., yeast or *E. coli* display), for intracellularly produced enzymes, full or partial cell lysis or secretion of the enzyme is necessary. Finally, intracellularly expressed protein can be screened without lysis when the substrate is transported in and the product out of the cell to be detected by a cocompartmentalized sensor strain.

The practical challenges for the expression system include the following: (*i*) *monoclonality*, expression from single variants, while also having to recover enough DNA for decoding to avoid the loss of hits (Figure 8); (*ii) access*, the need for the target enzyme to reach its substrate, i.e., not be physically separated by, e.g., a cell membrane; (*iii*) *sensitivity*, sufficient amounts of protein to turn over enough substrate to product to exceed the detection threshold; so expression systems have to be efficient.

### 6.1 *In Vivo* Expression

Bacterial lysates have been used most often for making protein available in droplets:^2,236−238^ the protein is produced, e.g., in *E. coli* that are grown offline (with the protein remaining in the bacterial cytosol) and compartmentalized into droplets, followed by cell lysis. If single bacteria are coencapsulated with a lysis agent and substrate, it is especially important that a high-copy-number plasmid is used to allow for efficient DNA recovery. High-copy-number plasmids are readily available and typically harbor inserts of 3–5 kb in length. This is optimal when screening for improved variants in a directed evolution project,^2,117,125,128^ but also functional metagenomic campaigns for the discovery of new enzymes from environmental DNA in plasmids have been successful.^118,196^ When larger inserts are screened, i.e., fosmids or cosmids (with 30–40 kb environmental DNA per vector), no high-copy-number constructs are available. The very low copy number of fosmids or cosmids requires amplification for successful recovery.^131^ To this end, single cells can be compartmentalized and then grown in droplets.^144^ Adding a level of control, an *E. coli* system has been introduced that allows for the titratable induction of lysis of a defined fraction of the bacterial population.^239^ Alternatively, after bacterial growth, complete lysis can be achieved by picoinjection of lysis agents.^144^ Avoiding the need for lysis, enzymes can also be expressed in the bacterial periplasm^80,240^ into which many substrates can diffuse, be displayed on the bacterial^120,241^ or yeast surface,^122^ or be secreted.^113,114,242^ In these four approaches, living cells are recovered after sorting, offering the possibility to enhance recovery by growth amplification.

Microfluidic assays with whole cells have also been successfully applied to the discovery of active catalysts.^243^ The screening of intact cells can be especially useful in metabolic engineering when entire pathways or different genomic locations are involved in the target phenotype, e.g., improved protein secretion.^112,113,115,116^ or the production of secondary metabolites combined with a sensor strain for detection.^225^

### 6.2 *In Vitro* Evolution

*In vitro* expression systems are an attractive alternative to cell-based screening systems. They either use the unpurified protein synthesis machinery of cells^244^ or a defined mix of purified components.^245^ Cell-free directed evolution campaigns have four key advantages: (i) they are unconstrained by transformation efficiency; (ii) they are unaffected by potential toxic side effects of the expressed protein to the survival of the host organism; (iii) they can be carried out under conditions that avoid biological (arising from the proteome of the host organism) and chemical background reactions (e.g., by changing to a nonphysiological pH); and (iv) they enable quick workflows not depending on cell-based library cloning. Indeed *in vitro* expression systems were already used in the first functional screening studies in polydisperse droplets targeting DNA modifying enzymes.^29,90^ In addition, there is the conceptual beauty of the droplet as an *in vitro* compartment that resembles artificially created protocells, as a vessel accommodating just one biochemical process that is to be evolved without interference from other processes.

On the other hand, practical challenges complicate *in vitro* evolution. Since monoclonality requires just one variant per droplet, DNA recovery can be difficult. Early studies reported successful enrichment of active library members from only one DNA molecule per droplet,^26,29,89^ or bead,^30,106^ but DNA recovery may be suboptimal. Emulsion PCR^231^ or rolling circle amplification (RCA)^150^ in droplets prior to expression is an option for amplification. Regarding workflow design, thermal cycling and the reagents required for RCA are incompatible with the available *in vitro* expression systems which must be built into later steps. For example, IVTT components were added via picoinjection^130^ or electrocoalescence of two droplets^150^ only after the DNA amplification step. This was achieved by Holstein et al.^121^ in a multistep workflow for the directed evolution of proteases that thus far is the only demonstration of screening of *in vitro* expressed enzymes in microfluidic droplets.

Experimental *in vitro* alternatives exist: the coding DNA, expressed protein,^106^ and products can, after initial droplet compartmentalization, be captured on a single bead^28,32,75,204^ to preserve the genotype–phenotype linkage. After de-emulsification and washing steps, the addition of chemicals in a solution and sorting by FACS can proceed without microfluidics, and the union of genotype and phenotype on a bead allows recovery and decoding of hits without compartmentalization.

## 7 Reaction Types Amenable to Microfluidic Enzyme Screening

The starting point of any directed evolution campaign is the availability of a robust assay that allows for accurate quantification of the reaction progress in each droplet. Tables 4 and 5 give an overview of the reactions currently amenable to droplet screening, covering all seven enzyme commission number (EC) classes (oxidoreductases, transferases, hydrolases, lyases, isomerases, ligases, and translocases). The criterion for inclusion in these tables is at least a successful enrichment in monoclonal format (one gene per droplet). Evidence of successful directed evolution experiments is indicated as the proof that single library members in a library of great diversity can be identified and recovered.

**Table 4:**
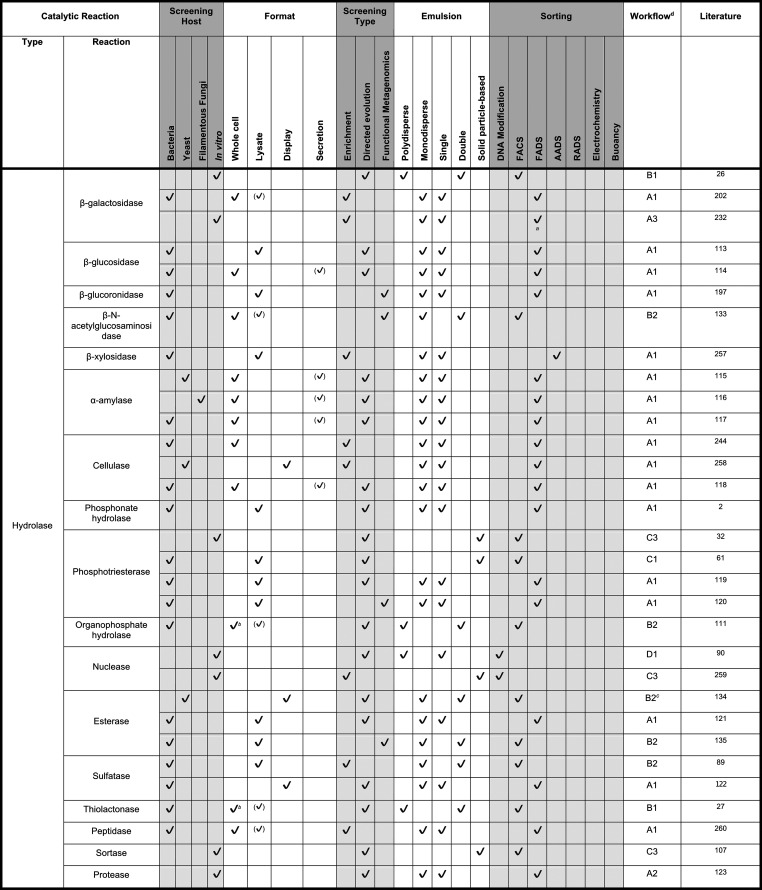
Enzyme Assays Demonstrated in Microfluidic Droplets Categorized by Reaction Type, Part 1^e^

a Fluorescence-activated electrocoalescence rather than FADS (i.e., a sorted droplet is merged into an aqueous stream for more efficient DNA recovery).

b Substrate added to oil phase and diffused into droplets and cross cell membranes or spontaneous lysis.

c In a variation to most other procedures, the second emulsification step is performed *before* incubation.

d Assigned worflows are discussed in section 8, Figure 9. Check marks in brackets indicate formats inferred from publication.

e Only assays in a monoclonal format that achieved at least enrichment are included.

**Table 5:**
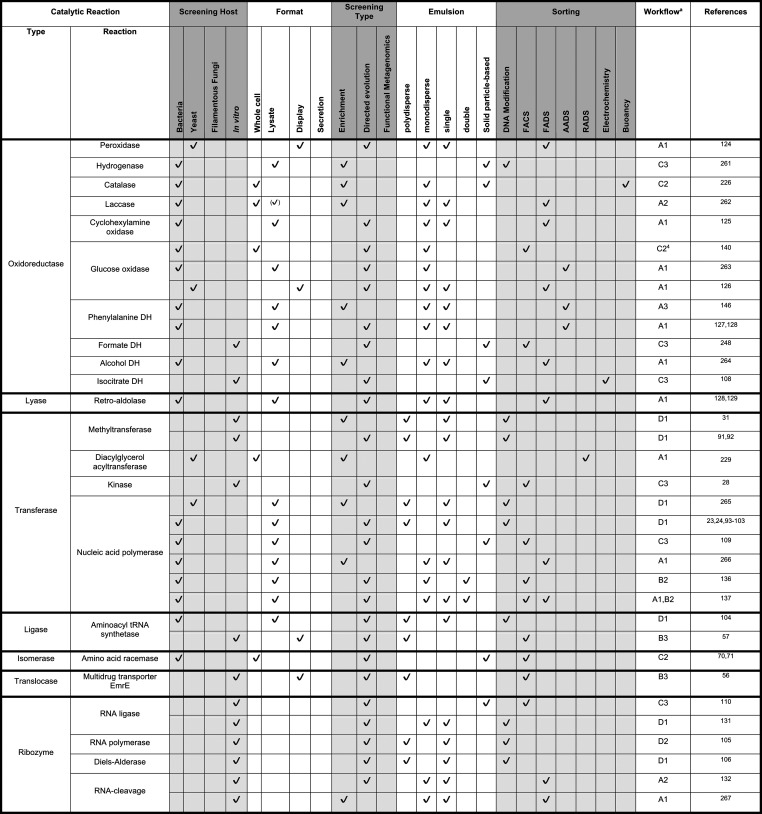
Enzyme Assays Demonstrated in Microfluidic Droplets Categorized by Reaction Type - Part 2^c^

a Assigned worflows are discussed in section 8, Figure 9. Check marks in brackets indicate formats inferred from publication.

b Type refers to enzyme classes, with ribozymes as a seperate category. EC classes are surrounded by bold frames. The remaining EC class “hydrolase” is covered in Table 4.

c Only published assays in a monoclonal format that achieved at least enrichment are included.

As in directed evolution, in general, many screening campaigns have targeted hydrolase reactions, for which fluorogenic or chromogenic substrates are readily available for the most straightforward way of following reaction progress by optical interrogation of droplets. Typically the natural leaving group is replaced by a fluorophore or chromophore, and the reaction product lights up: the hydrolyses of peptides, sugars, and carboxy-, phospho-, phosphono-, and sulfoesters have been assayed in this way. Such substrates have large optically active hydrophobic leaving groups, so the molecular recognition properties of such model substrates may be altered, and their typically higher reactivity (with leaving groups with lowered p*K*_a_ values compared to native substrates) makes observation of promiscuous reactions more likely. Alternatively, assays of proteolytic^121^ or glycolytic^113^ activity based on the autoquenching of BODIPY-labeled substrates that generate fluorescence after cleavage have also been successful. While chemically unactivated bonds are cleaved, the assay is not sequence-specific, reporting on activity rather than specificity.

For many relevant substrates, the cleavage of one particular bond does not directly result in the generation (or unquenching) of an optically active molecule. Coupled reaction systems that convert an optically inactive product into a downstream optical signal can potentially expand the scope of the assayable reactions. For example, free thiol groups produced by thiolactonase activity can be detected by fluorogenic compounds that react with the product thiol to form a fluorophore. Thioester hydrolysis can thus be followed by fluorescence without a custom-made substrate and without a potentially non-natural bulky leaving group.^27^ In more complex cascades, optically inactive reactants were coupled to downstream fluorescence^123^ or absorbance^125,126,144,256^ readouts via secondary reactions, covering redox reactions. Once reliably established, coupled reactions simplify the requirement for custom-made or expensive substrates that may only be available for standard reactions. Cascade reactions can be highly specific for the initial substrate (e.g., a natural sugar^256^ identified by a specific hydrolase, albeit without an optical signal), while the downstream reactions that process the initial product to create an optical signal are generic.^256^ In this way, the same assay mode can be used for a range of evolution campaigns. As long as high-quality enzymes with sufficient specificity for the first reaction are available, direct selection pressure can be applied e.g. to a range of natural substrates, with the same detection setup.

*In vitro* systems provide an avenue to set up product detection manifolds that would be hard to use in cell-based systems. A potentially generalizable platform has been developed for NAD(H)-utilizing enzymes, taking advantage of protein (and, in the future, nucleic acid) sensors for product detection. Here, highly functionalized microbeads were decorated with multiple copies of identical enzyme variant-encoding DNA on each bead,^246^ together with a bead-immobilized analogue of the cosubstrate NAD^+^. These beads were then compartmentalized in polydisperse water-in-oil emulsion droplets, where they were exposed to a cell-free expression mixture and enzyme substrate so that reaction progress (in this case by the model enzyme format dehydrogenase) led to a concomitant turnover of NAD^+^ to NADH. The addition of a fluorescent-protein-based sensor of NAD(H) then serves to report the redox state of the bead-immobilized cofactor, and flow cytometric sorting of beads identifies those with maximal reaction progress by sensing the ratio of NAD^+^:NADH on each bead.^247^ Reminiscent of earlier work,^30^ the beads constitute a genotype–phenotype linkage^248^ that is initially isolated by a droplet compartment and sorted after its removal on the basis of the distinguishing capacity of an added sensor. The more sensor molecules that become available,^249−254^ the more versatile this approach will be for future assay design.

## 8 Fully Integrated Workflows in Directed Evolution Campaigns: From Model Enrichments to Examples for Successfully Integrated Systems Validated by Library Screening

The availability of devices, analytical interfaces, a range of assays (with an understanding of their dynamic range and sensitivity), and proof-of-principle experiments is an important preliminary of setting up screening experiments (Figure 9). Enrichment experiments can help to assess whether a workflow is fit to operate and quantification of the observed enrichment is a helpful benchmark for iterative improvements. In enrichment experiments, a defined mix of positive and negative clones is sorted, and the amount of positive variants after sorting is assessed experimentally. There are two ways to calculate enrichments, different in how they define the fraction of positive clones before and after sorting.

**Figure 9:**
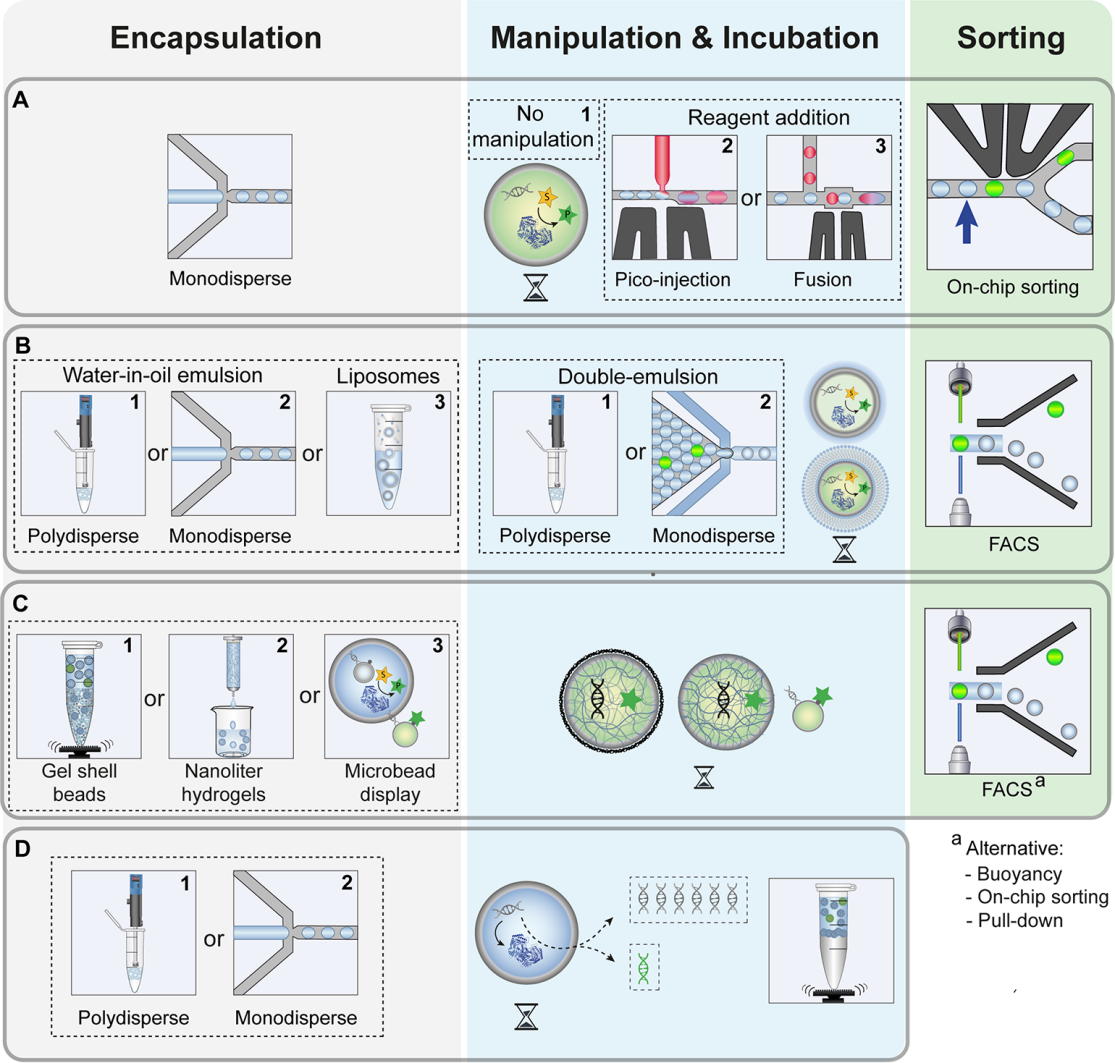
General workflows to screen for enzymatic activity in droplets. Subfigure numbering indicates workflows assigned in Tables 5 and 6. (A) Reaction in monodisperse droplets and droplet sorting. Monodisperse droplets are produced, incubated and analyzed either without manipulation (**1**) or manipulated by picoinjection (**2**) or droplet fusion (**3**). After analysis, droplets are sorted on-chip. (B) Double emulsions and liposomes sorted by FACS. Polydisperse (**1**) or monodisperse (**2**) droplets are produced and incubated. In a second step, a polydisperse (**1**) or monodisperse (**2**) double emulsion is formed and then sorted by FACS. An alternative to double emulsions is direct encapsulation in polydisperse liposomes (**3**) which can be incubated and sorted by FACS. (C) Solid-particle-based genotype–phenotype linkage. Gel-shell beads (**1**), nanoliter hydrogels (**2**) or microbeads (**3**) are produced, incubated, and sorted by FACS. Sorting has also been performed by buoyancy, pulldown, or on-chip droplet sorting if the solid particle remains encapsulated. (D) Selection of nucleic acid-manipulating enzymes by encapsulation without sorting. DNA libraries are compartmentalized in a polydisperse (**1**) or monodisperse (**2**) emulsions, and a readout is directly achieved by manipulation of the encoding gene (e.g., amplification). The droplet emulsion is broken and the activity of variants is represented by the quantity of its encoding gene.

Baret et al. define enrichment η as follows:^201^
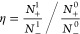
with *N*_+_^1^ representing positive clones after sorting (true positive), *N*_–_^1^ negative clones after sorting (false positive), *N*_+_^0^ positive clones before sorting, and *N*_–_^0^ negative clones before sorting. In contrast, Zinchenko et al. define enrichment η′ as the ratio of percentages of positive clones after and before sorting:^87^
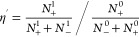


This can lead to large differences in reported enrichment factors (η and η*′*) as illustrated by the following example. If a 1:100 dilution is used in an enrichment experiment and after screening 95 positive and 5 negative clones are found, the enrichment calculated according to Zinchenko et al. would be η*′* = 95 and an enrichment of η = 1881 would be calculated according to Baret et al. These different ways of calculation need to be taken into consideration when evaluating reported enrichment factors.

However, the bar for a successful library experiment is higher still. Several additional challenges have to be met: (i) *Long-term operability*: Devices have to run for hours (instead of the few seconds of a movie that characterizes a device or module functionality) to screen an entire library. (ii) *Single-gene recovery*: In contrast to an enrichment experiment, where multiple copies of the positive model hit are supplied, libraries may contain just a few clones that satisfy the selection criterion. These have to be recovered efficiently to make the screen successful and represent the selection output faithfully. (iii) *Compatibility*: Modules developed in isolation have to be assembled to implement multistep workflows. For workflow design, the intrinsic throughput per time of individual module operations determines whether to develop continuous or discontinuous workflows (with the latter allowing more flexibility in the combination of modules). Practicalities (e.g., back-pressure and convenient operational control) will also be important considerations when modules are combined.

**Figure 10:**
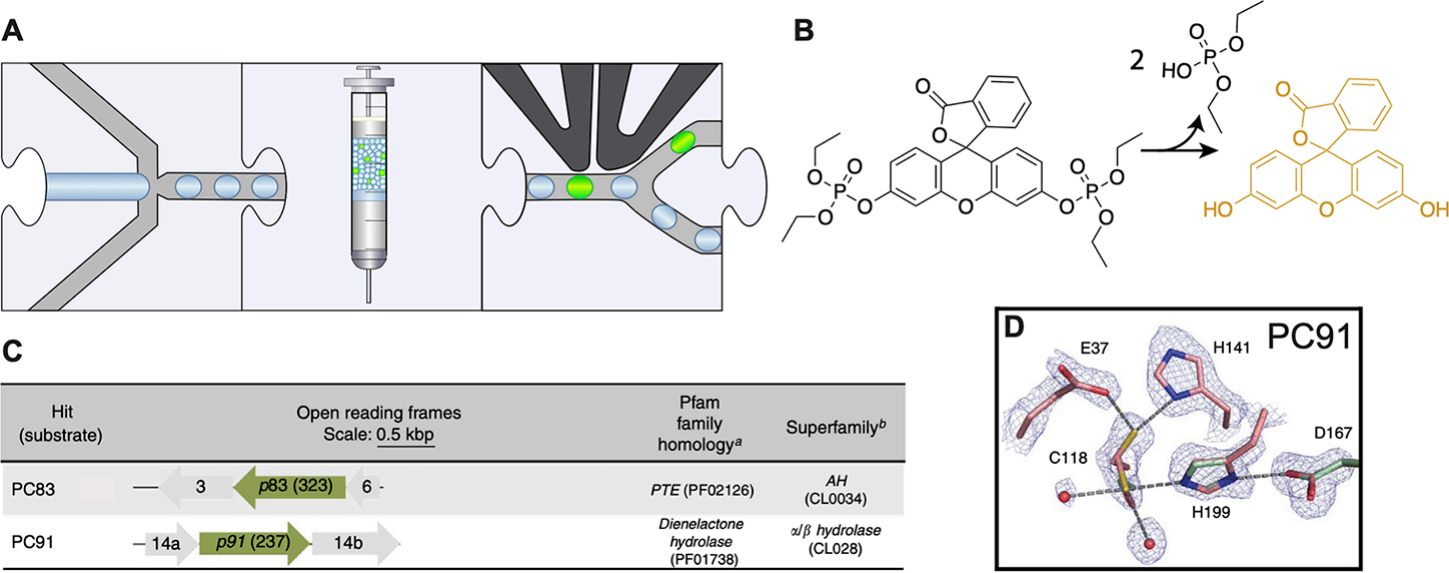
Functional metagenomic discovery of phosphotriesterases.^118^ (A) Workflow. Monodisperse droplets are generated with a metagenomic library expressed in an *E. coli* host and a fluorogenic substrate. The droplets are stored off-chip and then reinjected into a FADS device that sorts fluorescent droplets. (B) Fluorogenic assay. A fluorescein-phosphoester derivative is hydrolyzed to yield fluorescein that can be detected in FADS. (C) Hits from metagenomic screening. While PC83 was predicted to be a potential phosphotriesterase by Pfam domain recognition, the hit PC91 has open reading frames with Pfam family and superfamily assignments that had not been previously associated with triesterase activity. (D) Active site of the novel phosphotriesterase PC91 that uses a catalytic triad in its catalytic mechanism.

**Figure 11:**
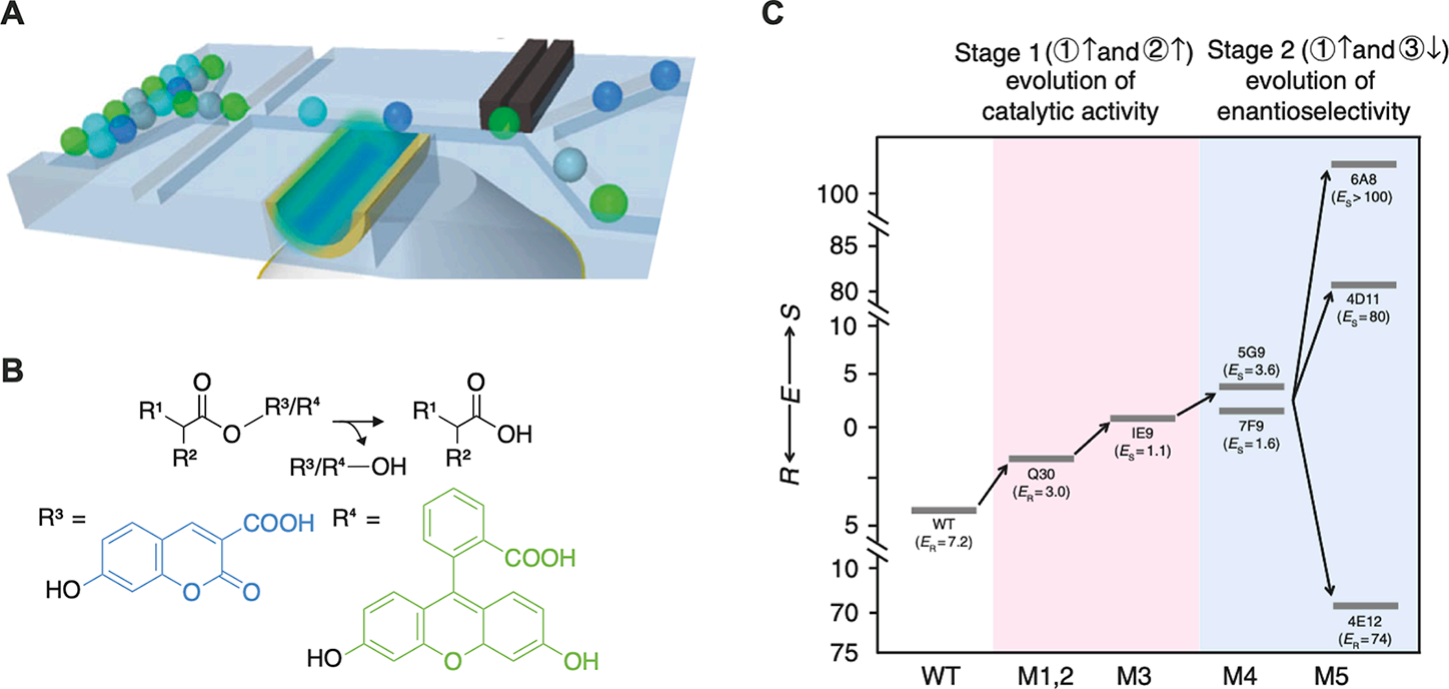
Directed evolution of an enantioselective esterase using a dual-channel device.^119^ (A) A FADS device allowing excitation with two lasers was designed to simultaneously report on the conversion of two different fluorophores (indicated by green and blue droplets), in a workflow similar to that in ref (128). (B) The profen ester substrate of the enzyme can be modified with either a coumarin or a fluorescein leaving group. Modification of different profen enantiomers with distinct fluorophores allows screening for enantioselective esterases using the dual-channel FADS device. (C) A profen esterase was evolved over multiple rounds of directed evolution. Cumulative improvements in enantioselectivity E are displayed for the wild type and variants arising from several rounds of directed evolution. First, a library was screened for general improvement of catalytic activity, without regard to enantioselectivity. This screen yielded Q30 (with a 2-fold improvement) and, after a further round of error-prone PCR, 1E9 (4-fold improvement) as the top performers. In stage two, the dual-channel device was used to gate for enantioselective variants, and variants 6A8 and 4E11, with 700-fold and 560-fold improved enantioselectivity, were identified.

**Figure 12:**
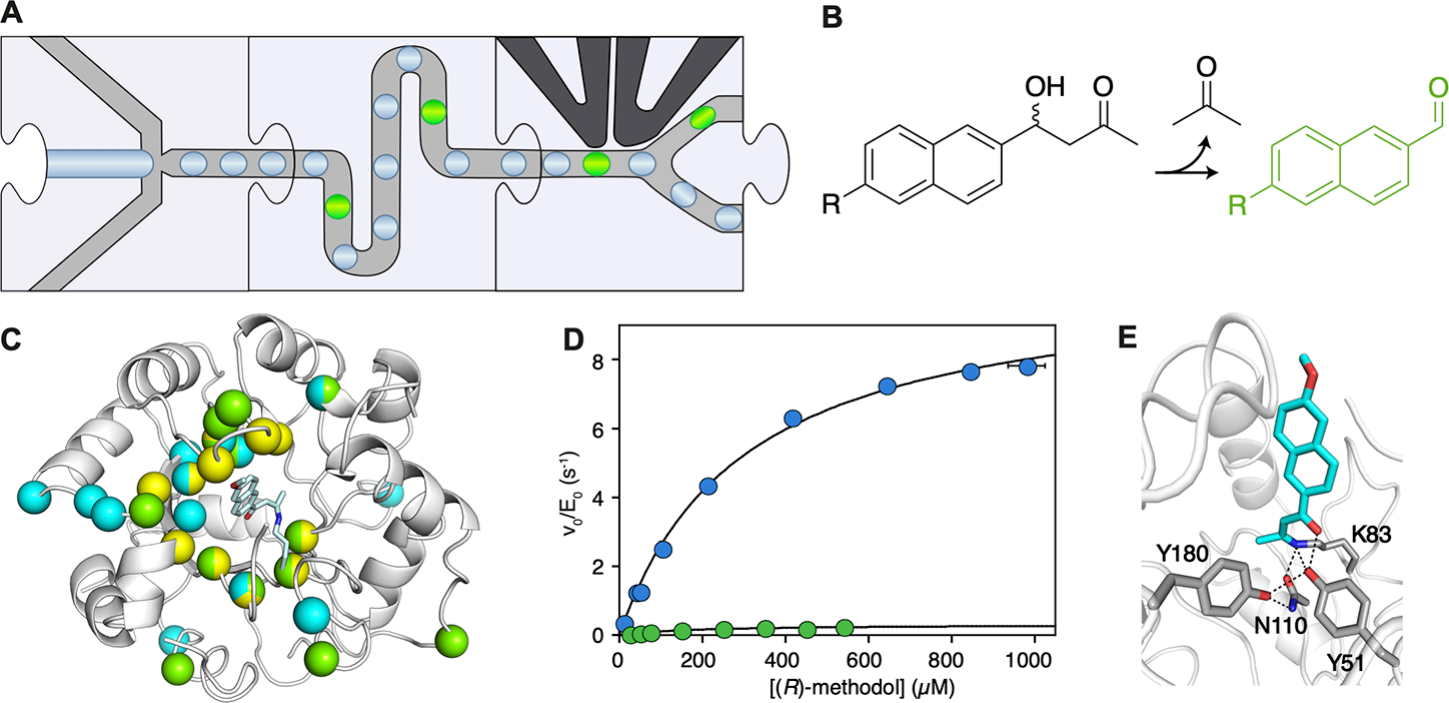
Directed evolution of an aldolase.^128^ (A) Workflow. Monodisperse droplets are generated, including the library expressed in *E. coli* and the substrate. The droplets are incubated on-chip to enable short incubation times. Fluorescent droplets are sorted. (B) Assay. The aldolase cleaves the substrate, releasing a ketone and a fluorophore. (C) 11 mutations (yellow spheres) were introduced to generate the starting point that was subsequently optimized by low-throughput directed evolution (green spheres) over 13 rounds of evolution followed by five rounds of directed evolution in droplets (cyan spheres). (D) Michaelis–Menten plot comparing the starting point of the ultrahigh-throughput campaign (RA95.5-8, green) with the variant with the highest activity (RA95.5-8F, blue) after directed evolution. (*E*( Catalytic tetrad emerging after directed evolution. We thank Prof. Donald Hilvert for providing the material for the subfigures C, D and E.

**Figure 13:**
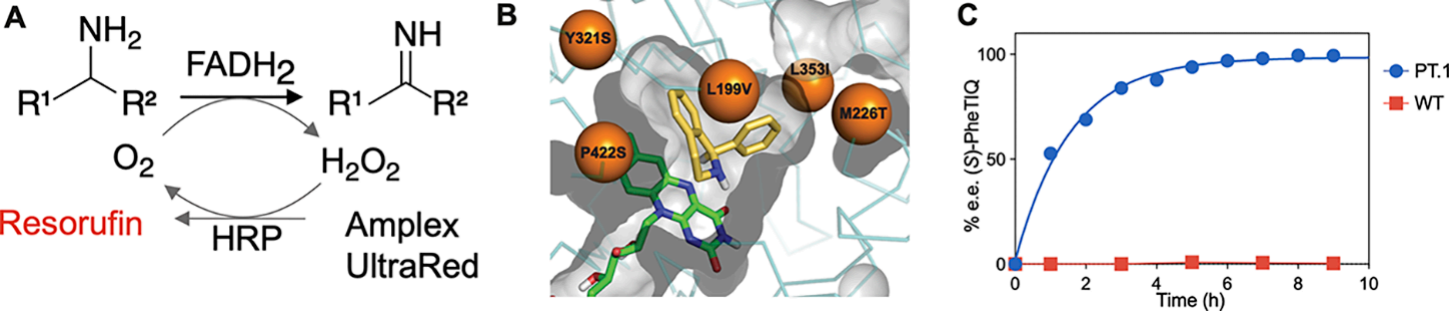
Directed evolution of an amine oxidase.^123^ (A) Coupled assay used to screen for amine oxidase activity. The amine substrate is oxidized, yielding H_2_O_2_ as byproduct, which oxidizes Amplex UltraRed to Resorufin. (B) After one round of directed evolution, the active site of the enzyme is remodeled by introduction of five mutations. (C) The variant yielded from directed evolution (PT.1, blue) has a 960-fold improved *k*_cat_/K_M_. We thank Prof. Donald Hilvert for providing the material for the subfigures B and C.

**Figure 14:**
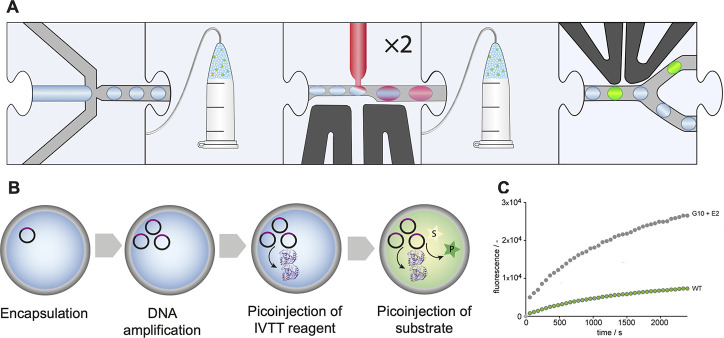
Droplet manipulation by picoinjection enables sequential addition of reagents.^121^ (A) Monodisperse droplets are produced and stored off-chip, followed by two picoinjection steps with incubation off-chip, and FADS. (B) The workflow enables stepwise DNA amplification by RCA, protein expression by IVTT, and conversion of substrate to generate a fluorescent readout using reagents that otherwise would be incompatible with each other. (C) The improved variant G10+E2 shows a 5.5-fold improved activity.

This is why the implementation of fully integrated workflows that have yielded genuine hits in library screening experiments is the decisive step en route to making universal use of droplets to find functional proteins. Figure 9 represents the patterns of workflows that have passed this test, and Figures 10–14 detail successful examples.

The first workflow (Figure 9A) summarizes a screen in monodisperse microfluidic droplets using assays with an optical readout, e.g., fluorescence or absorbance. A monodisperse emulsion is generated by compartmentalizing library members together with substrates. The emulsion is either incubated or directly screened using droplet sorting activated by a readout (e.g., FADS^201^). In addition, on-chip manipulation steps, including, e.g., picoinjection^121,130^ or droplet fusion,^231^ can be carried out prior to sorting.

An example of finding a “needle in a haystack” against overwhelming odds is the screen of a metagenomic library of more than a million members from various natural environments for a phosphotriesterase reaction, a hydrolytic reaction related to a non-natural substrate. Here monodisperse droplet generation was followed by incubation and FADS to screen for hydrolase activity (Figure 10A).^118^ A substrate generating fluorescence upon cleavage has been used (Figure 10B), and 8 phosphotriesterases (with a *k*_cat_/*K*_M_ = 9 × 10^5^ s^–1^ M^–1^ for the best one, PC83) have been identified. In addition to homologues of previously identified metal-dependent triesterases, the hit PC91 turned out to be a member of the α/β-hydrolase superfamily, with an esterase-like catalytic triad and without an active site metal (Figure 10C and D). PC91 is the first metal-free bacterial triesterase to be described and—when represented in a sequence similarity network—breaks new ground in unannotated regions of sequence space, showing that microdroplet-based ultrahigh-throughput screening of metagenomic libraries provides functional information that cannot be predicted. Finding such hits by sequence-based methods would not have been possible, as this type of enzyme had only been associated with carboxyester hydrolysis. Promiscuous activities such as this one are hard to predict, and hits are rare for non-natural substrates. This is to say that a screen of tens of thousands of clones in a robot would—statistically (based on the finding of 8 hits among 10^6^ library members)—only have been successful every 10th time: droplet technology was necessary to find *any* hits. The same assay has been used to further evolve PC91, yielding variants with a 400-fold increase in activity after only two rounds of directed evolution.^117^ Here, the initially discontinuous workflow was made continuous by the introduction of delay lines to account for the increased proficiency of the catalysts emerging from selection rounds, requiring incubation times of tens of minutes (rather than initially days).

In a further example of harvesting enzymes from the same metagenomic library using the workflow depicted in Figure 9A, a screen for β-glucuronidases identified a candidate for this particular activity in an unexpected sequence context, i.e., with neglectable homology to previously characterized enzymes with this function.^196^ While having little sequence homology to known β-glucuronidases, it was located in a glycosyl hydrolase family (as classified by CAZy) that had no recorded evidence of β-glucuronidase activity at the outset of this study but several other recorded activities.

Another workflow implementation in Figure 9A (monodisperse droplet generation, incubation off-chip, and FADS) was the work of Ma et al.,^119^ who engineered an enantioselective profen esterase. An innovative dual laser FADS device was used (Figure 11A) to monitor the turnover of two different fluorogenic substrates to screen for selective variants (Figure 11B). Multiple rounds of directed evolution gave a variant with 700-fold improved enantioselectivity.

Similarly, Obexer et al. used the workflow in Figure 9A to improve a previously optimized artificial aldolase 30-fold.^128^ Monodisperse droplets were incubated on a chip to enable short incubation times (Figure 12A). A methodol derivative that forms a fluorescent product upon reaction was used as the substrate (Figure 12B). The delay line was varied in length to reduce the incubation time from 1 h to 5 min. This controlled approach in delay line design allowed for the selection of increasingly more proficient catalysts during the campaign. After five rounds of directed evolution, the aldolase was improved 30-fold, salvaging a previously stalled directed evolution campaign (Figure 12C and D). Intriguingly, the evolution campaign yielded a completely remodelled active site with a new catalytic tetrad erasing the original catalytic apparatus (Figure 12E).

To engineer an amine oxidase, Debon et al.^123^ implemented a different assay within the familiar setup of Obexer et al. (Figure 12A).^128^ Coupled assays are far more versatile than direct assays, as they can be used for a broader range of target reactions. Additionally, they do not rely on mock substrates with bulky fluorogenic groups, allowing screening for authentic substrates used in the targeted application. In their assay, Debon et al. read out the production of H_2_O_2_ by the amine oxidase indirectly via oxidation of Amplex UltraRed to the fluorescent dye resorufin (Figure 13A). The identification of a mutant with a 960-fold improvement in *k*_cat_/*K*_M_ with a completely remodelled active site (Figure 13A and B) in only one round of screening demonstrates the potential of ultrahigh-throughput screening to improve biocatalysts in time scales compatible with the fast pace of product development in industry.

The previously mentioned formats rely on expression in cells and, therefore, cannot be used to engineer cytotoxic proteins. To engineer a cytotoxic protease, Holstein et al. developed a microfluidic workflow enabling *in vitro* expression of the enzyme (Figure 14A).^121^ Reaction conditions are complex (>70 components) and cannot be performed in one pot. To ensure compatibility of the reagents, DNA amplification by rolling circle amplification (RCA) is followed by two picoinjection steps used to sequentially inject IVTT reagent and substrate (Figure 14B). Directed evolution (based on focused libraries followed by their reshuffling) using this workflow yielded Savinase variants with up to 5.5-fold improved activity (Figure 14C). This evolution experiment would not have been possible in *E. coli.* (Indeed, the resulting variants had to be expressed in *B. subtilis* to obtain sufficient quantities to be characterized.)

The more accessible, “democratic” format of double-emulsion droplets (water-in-oil-in-water) is shown in the workflow in Figure 9B, where flow cytometric sorting in a FACS replaces on-chip FADS. Both initially poly-^77^ and monodisperse^87^ droplet formats have been used for screening of libraries from environmental^131,133^ or randomized^26,27,109,132,134^ origins. Similarly, liposomes can be used for encapsulation, followed by screening using FACS. This has been successfully applied for the directed evolution of β-glucoronidase,^110^ aminoacyl-tRNA synthetase,^55^ and the multidrug transporter EmrE.^54^

Another innovative workflow in microfluidics-based ultrahigh-throughput screening for enzyme activity employs immobilization on solid particles (beads)^28,30,59,68,69,105−108,138,225,247,258,260^ and is shown in Figure 9C. A variety of different systems have been used in a fashion compatible to enzyme engineering. (i) Agarose beads coated by a polyelectrolyte complex around the core (gel-shell beads) retain small molecules that can be used as a readout in FACS and the enzyme-encoding gene. This system has previously been used in the directed evolution of phosphotriesterase.^59^ (ii) Another technique to couple genotype and phenotype is based on monodisperse nL-sized hydrogels that can be formed by laminar jet breakup.^66^ Hydrogels couple genotype and phenotype, for example, by retaining a fluorescent bacterial host,^68,69^ enabling sorting by FACS or by gas formation inducing a density shift.^225^ (iii) Reaction partners can also be displayed on DNA-carrying microbeads enabling the coupling of genotype and phenotype (microbead display). For enzyme engineering, microbead display has been pioneered in the directed evolution of phosphotriesterase^30^ and has been used in modified formats for screening for kinase,^28^ dehydrogenase,^106^ nucleic acid polymerases,^107^ RNA ligase,^108^ hydrogenase,^260^ and sortase^105^ activity.

**Figure 15:**
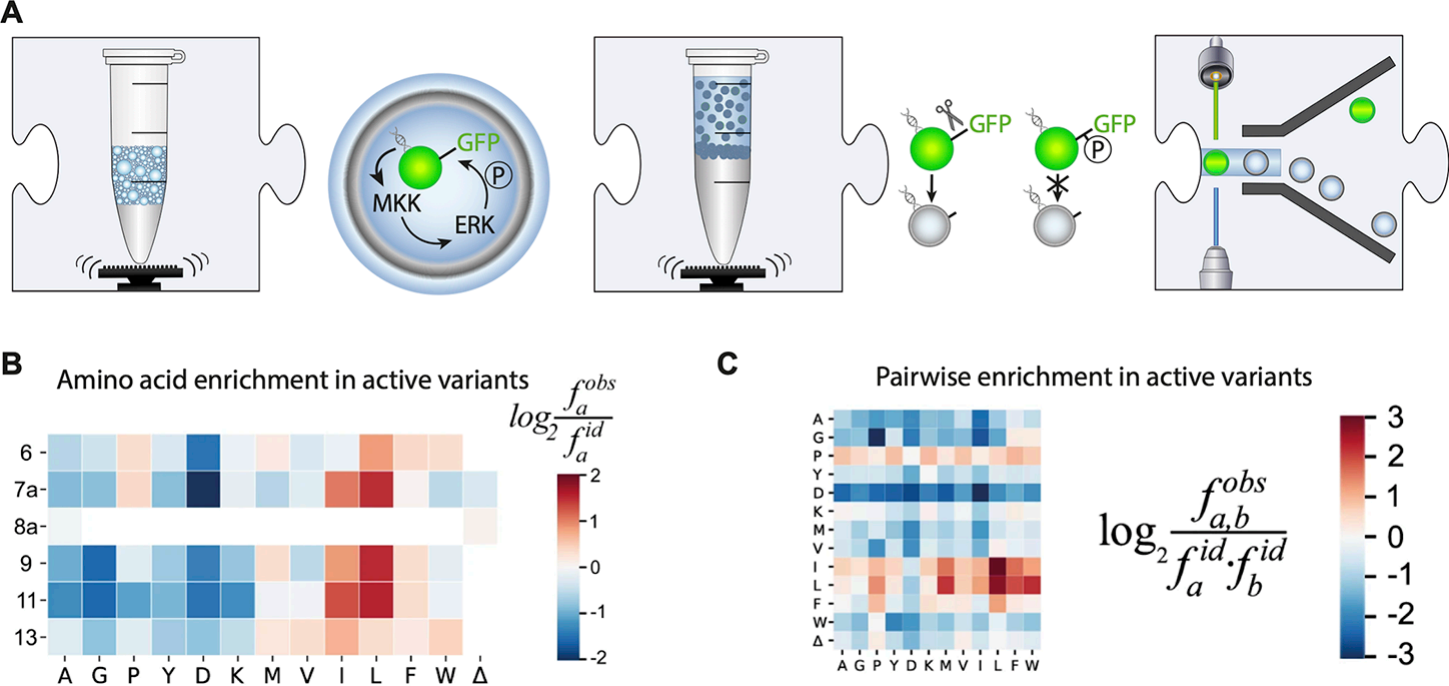
Paramagnetic bead-based kinase screening platform.^28^ (A) Screening workflow. Beads carrying an SpliMLiB MKK library are encapsulated into a polydisperse emulsion. The beads also carry GFP that is coupled to the bead via a peptide sequence that serves as a recognition motif to chymotrypsin and can be phosphorylated by ERK. *In vitro* transcription and translation are used to express MKK from the library, which then activates ERK by phosphorylation. After de-emulsification, beads are treated with chymotrypsin. Beads carrying GFP with a phosphorylated linker (encoding active MKK1) are resistant to proteolysis and so remain GFP-labeled and can be sorted with ultrahigh-throughput with FACS. (B) Enrichment in the active variants. Enrichment of the observed frequency (*f*^obs^) vs expected frequency (*f*^id^) is calculated for each amino acid at each position as a proxy for fitness. Large hydrophobic amino acids (especially leucine and isoleucine) are enriched at nearly all tested positions. (C) Pairwise enrichment. Enrichment of the observed frequency (*f*^obs^) for each double mutation over the expected frequency calculated from single-point mutation data. Mutation to leucine and isoleucine serves as the anchor allowing mutation to nonpreferred amino acids by exhibiting positive epistasis.

Bead-display-based screening has also been adapted by Scheele et al. to disentangle the encoding of substrate specificity in kinases.^28^ The encoding DNA of a kinase (MKK1) library is generated on a bead,^246^ encapsulated into a polydisperse emulsion, and expressed using IVTT (Figure 15A). Functional kinases then activate purified ERK2 by phosphorylation. The bead also harbors GFP that is immobilized with a linker peptide containing a serine residue that ERK2 phosphorylates. The emulsion is broken, and the beads are treated with chymotrypsin which only cleaves the non-phosphorylated linker. The beads are then sorted by FACS and NGS is used to correlate cascade activity and the encoded kinase gene. Thereby the fitness of 5 × 10^5^ independent variants was determined, and large hydrophobic residues were identified as a core feature of the MKK1 docking domain (Figure 15B). Additionally, substitutions to large hydrophobic residues exhibit pervasive positive epistasis, widening the available D-domain active sequence space and generating evolutionary contingency.

The seminal demonstrations of *in vitro* compartmentalized screening were evolution campaigns for DNA modifying enzymes. The corresponding schematic workflow is shown in Figure 9D and relies on self-modification of the *in vitro* compartmentalized gene. For example, methyltransferases were evolved that rendered their encoding genetic element resistant to restriction digest.^29,89^ Beyond that, ribozymes catalyzing RNA ligation^129^ and nucleases^88^ have been engineered. *In vitro* compartmentalization (IVC) has also been modified to engineer Diels–Alderase ribozymes by a physical linkage between the gene and the substrate.^104^ Perhaps the most robust example of this workflow is compartmentalized self-replication (CSR), which has been used extensively for engineering nucleic acid polymerases.^23,24,91−101^ CSR can also be coupled to other enzymatic activities in an approach called compartmentalized partnered replication, which has been used to engineer yeast tryptophanyl synthetase.^102^

**Figure 16:**
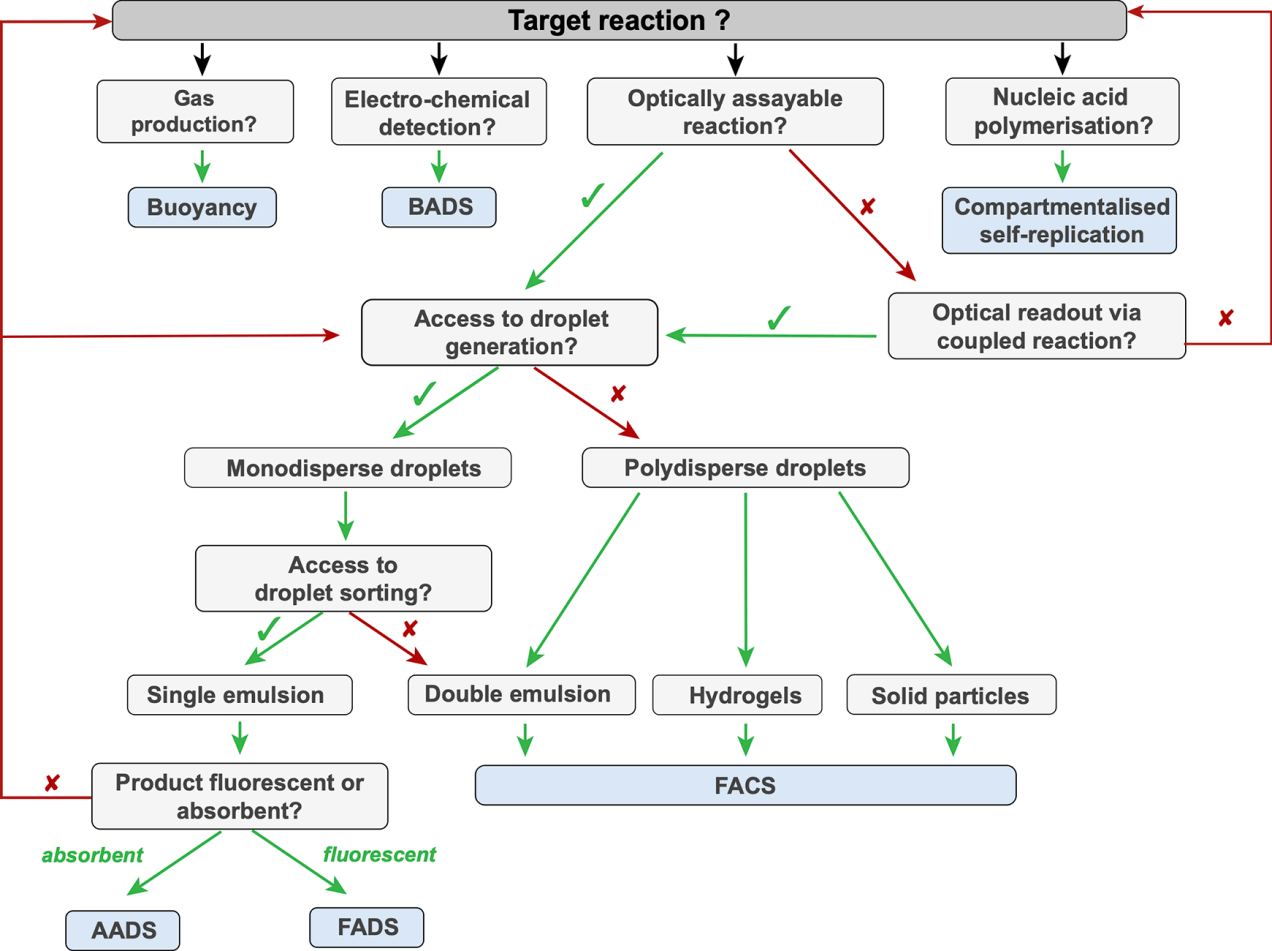
Decision tree for planning of microfluidic droplet assays according to a target reaction. This chart illustrates the choices that can be made when designing screening assays for microfluidic droplets and highlights the paths to sorting in different droplet formats, optical interrogation or reaction progress, and corresponding analytical interfaces. A successful demonstrated monoclonal enrichment experiment is the requirement for inclusion in this decision tree.

To facilitate custom workflow design for future droplet-based enzyme assays, we summarized relevant considerations in a decision tree (Figure 16) that guides the experimentalist from target reaction to assay type, droplet format, and sorting.

## 9 Troubleshooting

The successful examples of droplet-compartmentalized library screening experiments for directed evolution and functional metagenomics discussed in the previous section suggest that several complete workflows are in principle ready to be used by a wider audience. To make this happen, it will be important to understand the day-to-day troubleshooting that made the implementation of these examples successful. Interdisciplinary challenges can arise at several unfamiliar fronts, including emulsion and colloid science and their compatibility with biological processes (and cross-compatibility of biochemical reagents). Likewise, complex biological processes must be compatible with each other. Here, we discuss practical protocols to address implementation problems and facilitate or rescue experimental campaigns (Table 6).

**Table 6:** Troubleshooting Tips and Tricks for Microfluidic Experiments^a^

**Observation causing problems for high-throughput microfluidic screening experiments**	**Tips and Tricks**
**Running a microfluidic device**	
	
Droplets split after the formation junction or a jetting regime is reached	→ Reduce flow rate
Satellites or small droplets formed	→ Reduce surfactant concentration
Droplets merge after generation	→ Reduce flow rates
	→ Increase the surfactant concentration
Tubing does not stay in the device	→ Ensure tubing size is correct
	→ Check for blockage in the channels
	→ Check flowrates for pressure (e.g., μL/min vs μL/h)
Aqueous stream pulses away from channel edge	→ Ensure hydrophobic or hydrophilic coating is uniform
Aqueous stream pulsing irregularly	→ Check for air bubbles along the tubing and in the device
	→ Check for blockage
	→ Ensure the flow rates are not too low for the pump
	→ Ensure tubing is not too long (pressure increases with length of tubing)
Fibers arriving with the oil	→ Add filters to the device design (has become standard to help reduce blocking of the inlet)
	→ Filter all solutions before droplet generation
	→ Flush tubing to prevent microfibers/particles
Dust in a channel	→ Attempt to run the phase of that channel at higher rate and wait for dust to slowly move
	→ Press up and down on top of the PDMS to try to dislodge smaller particles
	→ Remove the tubing of the closest inlet/outlet to suck the dust/cells out. Follow this by backflushing from another inlet/outlet in the opposite direction, so that the dust moves out of the device
	→ Change device. It is often best to just start with a clean chip, as flushing and pressures can delaminate the chip
Droplets are not being made, and one of the phases cannot be seen	→ Check all connections for evidence of leaking
	→ Check for delamination
	→ Check for air bubbles along the tubing and in the device
**Collection of droplets**	
	
Droplet generation is unstable upon addition of outlet tubing	→ Inserting tubing increases the pressure in the device geometry
	→ Wait a few seconds for the flow to stabilize
	→ Reduce length of the outlet tubing (back-pressure increases as tubing length increases)
**Incubation**	
	
Droplets merge after incubation	→ If droplets are incubated in a collection tube or syringe, the emulsion at the top can get dehydrated and start breaking, causing merging
	o Add mineral oil layer on top of the emulsion (if using fluorous oil) or make droplets in mineral oil
	o Incubate within a humidity chamber
	→ Increase surfactant concentration
	→ If droplets are incubated in a closed chamber:
	o Check for air bubbles
	o Use anti-static gloves
Substrate or product leakage	→ Check background reaction leaking of substrate or product:
	o Test with equal volumes of your chosen buffer and max concentration of Substrate or Product to generate droplets
	o Incubate and image or analyze in flow cytometry
	→ Vary the concentration of surfactant
	→ Vary the oil/surfactant combination
	→ Chemically modify the substrate (e.g., more charge reduces leakage in fluorous oils)
	→ Addition of detergents and other additives may affect the stability of the droplets: assess droplet composition
Droplet shrinkage	→ Store droplets in oil or water
	→ Match osmolarity of the droplets and dispersed/carrier phase
	→ Cover HFE-oil droplets with mineral oil to avoid evaporation
**Re-injection of droplets into other devices**	
	
General instability of droplets at the inlet	→ Use anti-static gloves or trigger an anti-static gun across the collection chamber and tubing
	→ Ensure there are no vibrations disturbing the setup
	→ Rinse tubing and chamber with the carrier phase to remove microfibers and dust
Droplets are not packed	→ Packing of droplets is crucial to downstream processes (double-emulsion, picoinjection, sorting)
	→ In syringes: add a mineral oil layer on top of the emulsion in order to push droplets
	→ In tubing: add an air plug in tubing between the emulsion and an oil phase to help pack the droplets
	→ In chambers: add a fluorous oil (more dense than aqueous droplets) in the collection chamber so that droplets settle at the top of the chamber, ready for re-injection
Droplets are unevenly spaced for further manipulation	→ Reduce the width of the re-injection channel before the spacing oil so that droplets arrive single file
	→ Increase flow rate of the carrier phase to space out the droplets
	→ Pause the re-injection for a few moments before restarting
	→ Ensure the droplets are the correct size for the sorting device geometry
**Picoinjection**	
	
Satellites form after the electro-coalescence	→ Reduce surfactant concentration
	→ Reduce flow rates
	→ Increase the spacing between droplets
	→ Vary flow rates of the injected phase to match the timing of incoming droplets
	→ Decrease the voltage of the electric field
	→ Check voltage frequencies and pulse delay
Droplets merge or split upon picoinjection	→ Decrease the voltage of the electric field
	→ Check voltage frequencies and pulse delay
	→ Build a “Faraday moat” or ground electrode upstream and downstream of the electro-coalescence area
**Sorting**	
	
Droplets merge or split at the electrodes	→ Decrease the voltage of the electric field
	→ Check voltage frequencies and pulse delay
	→ Build a “Faraday moat” or ground electrode upstream and downstream of the electro-coalescence area
Signal not detectable over droplet background	→ Add a compound to the droplet mixture to offset the droplet background signal
	o Absorbance: any compound with the same absorbance wavelength will bring the signal into range (e.g., above/below the signal)
	o Fluorescence: droplet signal can be very close to oil signal levels so a μM range makes “empty” droplets detectable, to help determine the sorting threshold
Droplets are not sorting	→ Check that the electrodes are working by manually triggering the electrodes and determining whether droplets are pulled into the correct channel
	→ Check that there are no salt crystals in the electrode channel (if using salt electrodes)
	→ Check for air bubbles in the electrode channel or tubing
	→ Check for delamination or leaking between electrode or any potential area where short circuiting might occur: ensure that the metal or salt circuit is isolated
Droplets are not sorting into the correct channel	→ Add a bias oil inlet to steer droplets into the waste channel
	→ Equalize lengths of tubing to the (+) sorting and waste channels to ensure even pressures
	→ Raise tubing of positive outlet to prevent false negatives and/or increase length of positive outlet tubing
	→ Change frequency, voltage, pulse width, and delay of the electrical signal
**Recovery by transformation**	
	
Fewer variants recovered than expected	→ Use low-binding collection tubes and tips
	→ Flush (+) sorting channel collection tubing well (with nuclease-free water for genomic recovery)
	→ Supplement droplet content with EDTA (to avoid that long incubation times can lead to DNA degradation by metal-dependent nucleases)
	→ Use ultracompetent *E. coli*
	→ Add junk DNA (e.g., salmon sperm DNA) during extraction to reduce adsorption of recovered DNA to tube and tips
More recovered variants than expected	→ Ensure that the droplet sorting process was correct (see above), e.g. by inspecting the recorded video trace
	→ Reduce potential for contamination during the recovery process

a A practical guide for droplet generation, manipulation, sorting, and DNA recovery.

### 9.1 Challenges to the Integrity of the Droplet Compartment

Maintaining the integrity of the droplet is crucial for the duration of a screening experiment and requires a stable emulsion formulation. First, genotype and phenotype must remain co-compartmentalized to be able to decode individual hits after sorting. Second, the optical label must not escape from the droplet, as the sorting decision is based on a direct or indirect product concentration measurement. Indeed, product leakage between droplets would blur the distinction between “hit” droplets and those without an active clone and thus endangers the success of the experiment and so must be avoided. Substrate leakage into the oil phase can also be a problem, in which case the continuous supply of the (hydrophobic) substrate through the oil phase can be considered.^267^

These two requirements can often conflict, so exploration of various surfactant/oil combinations has been necessary to develop workable protocols that avoid coalescence of droplets (even when handled offline), minimize small molecule leakage, and stabilize the droplet compartments sufficiently to allow screening at the temperatures envisaged for the biocatalyst.

Stability and small molecule leakage unfortunately tradeoff against each other, so careful optimization of the type of oil/surfactant mixture is important, as well as their ratio and absolute amounts. Stability is easily satisfied e.g. by well-established emulsion oil/surfactant mixtures formulations with mineral oil and nonionic emulsifiers (e.g., ABIL90)^151,152^ or surfactants soluble in organic (e.g., Span80) or aqueous phases (e.g., Triton X-100, Tween 20/80). Lower emulsifier concentrations^268^ and additives (e.g., bovine serum albumin^152^ or cyclodextrin)^269^ help to establish sufficient fluorophore retention on time scales of hours. The use of inert perfluorocarbon carrier oils,^270^ together with fluorinated triblock surfactants,^271^ promised to abolish leakage (including between double emulsion droplets)^272^ based on the idea that a fluorous “third” phase with hydrophobic *and* lipophobic properties would not be attractive for small molecules. Fluorous oils should minimize leakage by offering only weak hydrogen bonds to fluorine for polar molecules (compared to water) and also be too polar to attract hydrophobic molecules. However, this has not been sufficient to abolish leakage problems. The addition of sugars to the aqueous phase has been shown to reduce leakage of resorufin, fluorescein, and coumarins across the mineral oil/Span 80 phase.^273^ The use of the fluorous oil FC-40 slowed down leakage of resorufin, albeit at the cost of emulsion quality.^150^ However, leakage still occurs, presumably because the exit of small hydrophobic molecules out of the aqueous droplet is entropically driven (restoring the disorder in water after removal of its local structuring around the hydrophobic solute molecule), even if there is no enthalpic gain upon arrival in the fluorous phase (with a lack of attractive interactions). Nevertheless, combinations of fluorous oils and fluorinated surfactants are now widely used also because they compare favorably in terms of stability and viscosity (lower than mineral oil).

The problem that hydrophobic small molecules are prone to leakage is general, but the *extent* of this effect is difficult to predict and must be experimentally determined (e.g., by microscope imaging or fluorescence measurements on chip^152,268,274,275^ or using oil-based flow cytometry).^269^ A straightforward leakage assay involves visualization of two populations of droplets, of which one contains the detected substance and is mixed and incubated with droplets without the analyte. Histograms are recorded at various incubation times to investigate the concentration change between the two droplet species.^152^

Modification of initially hydrophobic product (or substrate) molecules with charged groups helps to increase retention.^128,243,274−276^

The surfactant itself plays a role in facilitating leakage and maintaining stability. Higher surfactant concentrations increase stability but also promote leakage. Table 7 lists commercially available surfactant preparations, but some of them suffer from batch-to-batch variation and different degrees of purity. Detailed synthetic procedures have become available and will facilitate custom synthesis. Published syntheses e.g. of di- and triblock fluorocarbon surfactants^277^ make these reagents available in the absence of a commercial supplier. New surfactants are emerging, e.g., silicone nanoparticles (modified with 1H,1H,2H,2H-perfluorooctyltriethoxysilane, FAS), that form stable Pickering emulsions with reduced leakage of hydrophobic molecules^278,279^ or glycerol-based fluorosurfactants for better thermostability^280^ and reduced leakage.^281^

**Table 7:** Commercially Available Surfactants^a^

Commercial name	Vendor	Cognate oil phase	Features	Origin	Examples for use in protein engineering
FluoroSurfactant 008	RAN Biotechnologies	HFE 7500, FC-40	Most frequently used, allows for generation of stable droplet emulsions for various applications	Commercial based on ref (271)	(2,122,123)
Pico-Surf	Sphere Fluidics	HFE 7500, FC-40	Similar properties to FluoroSurf 008	Commercial	(126)
dSurf	Fluigent	HFE 7500	For ddPCR, single-cell analysis and cell culture. Allows use of lower pressures for PDMS chips	Commercial	NA
FluoSurf	Dolomite, Emulseo	HFE 7500	Compatibility with a broad range of reagents in the droplet phase. Fast droplet formation rate	Commercial	(240)
Droplet Generation Oil for Probes	Bio-Rad	Ready mix, can be diluted with HFE-7500	For ddPCR with probes, frequently used for other applications		NA
QX200 Droplet Generation Oil	Bio-Rad	Ready mix	For ddPCR with Eva Green dye, reduced leakage of hydrophobic compounds	(292)	NA
Fluoro-Phase	Dolomite	Ready mix, can be diluted with HFE-7500	Substantially decreased leakage, Pickering emulsion with lower stability	(278,279)	NA
1H,1H,2H,2H-Perfluoro-1-octanol	Sigma Aldirch, Alfa Aesar, other general chemistry vendors	HFE-7500, FC-40	Used to break emulsions, but also prevents wetting in the application with trains of noncontacting nano- or microliter droplets flowing along a channel	Commercial	(293)
					(294)
					(83)
Span 80/Tween 80/Triton X-100 mixtures	Fluka, Sigma Aldrich and other vendors	Mineral oil	Used to stabilize polydisperse droplets	(29)	(23)
					(295)
Abil Em 90 or Abil WE09	Evonik	Mineral oil, silicone oils	Stable emulsions with mineral oil, also used to generate polydisperse emulsions	(296)	(107,129,151,152)

a NB: We excluded oil mixes that cannot be purchased separately (e.g. the oil in 10X Genomics kits, where reagents are only available as a part of a larger kit).

No universally accepted model for the molecular mechanisms of leakage exists that would allow prediction of leakage properties from the structure, but hypotheses include diffusive models and the involvement of submicrometric vesicular structures.^282^ While these models are further refined, quantitative empirical insight into the leaking properties of oil/surfactant combinations^272,283^ will be valuable, and finally their biochemical compatibility has to be tested (e.g., with *in vitro* expression).^32^ In the absence of a predictive framework, iterative optimization of oil and surfactant combinations is necessary, as exemplified by Debon et al.^163^ in a survey of oil/surfactant combinations and their effects on droplet confinement and leakage, shrinkage, and tertiary phase formation.

Interaction with the chip material can affect the droplet contents and properties. PDMS conducts gases (air and water) so it can “dry out” droplets, leading to droplet shrinkage and formation of a solid structure that retains the droplet morphology unable to be retrieved.^151^ Storage in a closed system reduces droplet evaporation: sealing the inlet and outlet of a chamber device,^151^ covering the droplets with mineral oil,^2,87^ integrating a continuous water supply system into the chip,^284^ or containing droplets in a closed chamber^121,131^ helps to keep these effects under control. PDMS can also absorb^285,286^ or transport^287^ small molecules, suggesting a change of the chip material.^286,288^ Finally the coating of the chip, i.e., surface modification for hydrophobic or hydrophilic coating to match the carrier phase, choice of oil,^15^ or silanization of the PDMS devices (to reduce wetting effects or friction at the channel walls),^163^ can be considered.

### 9.2 Sensitivity

The assay sensitivity is, on the one hand, determined by the sensitivity of the detection method (Table 3). Yet, in a biochemical context, the background can also play a role: for example, in experiments with cell lysates, naturally occurring reactions such as carbohydrate-active enzymes^196^ can collectively bring about a background activity that rivals the activity of the library member. For cell-based screening, phenotypic variation can play a prominent role (10-fold variation across a cell population), especially when high-copy-number plasmids (advantageous for recovery, see below) are used. Especially for metagenomic selections (with enzymes cloned in suboptimal position with respect to a promoter), weak expression is likely, and the narrow difference between signal and noise can make it hard to identify candidates. In this case, lowering the selection threshold near the background, so that oversampling can be followed up by re-screening in plates with a reasonable (1:10 to 1:100) chance to detect a hit, can be helpful.^196^

### 9.3 DNA Recovery

Selecting a library member for its functional properties only provides molecular insight when its DNA sequence can be elucidated. This is nontrivial because the Poisson distribution with which each droplet experiment starts dictates just one type of DNA species per droplet. Therefore, the challenge is to amplify selected clones and decode the protein sequence on the basis of its DNA. Several strategies are possible:

(i) *Growth amplification in droplets*. Cells are compartmentalized as single entities but left to grow in droplets. Lysis is triggered by the addition of reagents by picoinjection, and an assay is carried out. Having more cells also leads to more enzymes, so the sensitivity of the functional assay is increased, while phenotypic variation is minimized.
(ii) *DNA amplification in droplets*. Especially for *in vitro* selections, where one DNA copy is compartmentalized, rolling circle amplification^121,189^ and isothermal amplifications^289,290^ and emulsion PCR^231^ are attractive and would also increase protein expression (by providing more templates) as well as more recoverable DNA.
(iii) *Growth after recovery*. When cells survive the assay, they can be regrown to ultimately produce enough DNA for sequencing. This can be achieved by in-droplet growth followed by partial lysis of cells (leaving enough cells to be recovered),^131^ by triggering partial lysis with a kill switch,^239^ or by avoiding lysis altogether in display systems (on yeast^122^ or *E. coli*(120)).
(iv) *Use of high-copy-number plasmids*. Near-perfect recovery (80%) can be achieved by employing high-copy-number plasmids in *E. coli*.^2,118,196^
(v) *Postselection PCR*. If very small quantities of DNA are recovered, their amount may preclude direct sequencing, but an amplification step recovers these. However, at the same time bias during the amplification may misrepresent selection outcomes, which will reduce the diversity of the recovered clones.

### 9.4 Uniformity of Droplet Operations in Long-Term Experiments

The premise of quantitative selection in directed evolution experiments is crucially dependent on producing identical droplet compartments, even over the hours that are necessary to reach millions of droplets. A range of practical problems can stand in the way—delamination of the PDMS chip, blocking of channels by dust particles, and uneven flow rates that lead to discontinuities are just a few examples. Table 6 summarizes these small but often annoying problems related to running microfluidic devices along with remedies.

## 10 Characterization

While the distribution functions obtained after sorting report on the kinetic profile of the library and the selected catalysts, further characterization is necessary (e.g., by measuring initial rates of product formation). Returning to the microtiter plate for this characterization is slow and cumbersome. Staying in a miniaturized format saves reagent volume and allows obtaining kinetic data for larger collections of mutants that are expected when ultrahigh-throughput screening is applied. More clones can be characterized in meaningful detail to draw up sequence- or structure–activity relationships and uncover mechanisms. The obtained kinetic data traces will also be useful for future modeling efforts when added into databases like EnzymeML.^297^ In addition to recording steady-state (Michaelis–Menten) or pre-steady-state kinetics, probing the acceptance of alternative promiscuous^194^ substrates, the effects of inhibitors, and the temperature stability of newly identified enzymes will be instructive. Sequence–function studies will greatly benefit from such quantitative insights, and their future combination with structure prediction from deep learning approaches^298,299^ should provide renewed impetus for protein engineering, perhaps even allowing for the reliable prediction of function.

Many different microfluidic systems for the quantitative measurement of kinetic or biophysical data have been devised (Tables 8–10). Concentration gradients have been generated in capillaries prior to droplet formation,^300−302^ by merging droplets,^82,303,304^ by variation of flow rates in the supply stream,^305^ or by continuous variation of the substrate concentration in the source well while making droplets.^83,306^

**Table 8:**
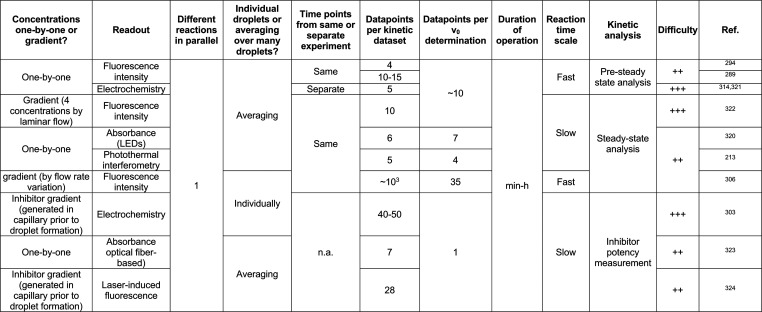
Microfluidic Systems for Kinetic Analysis Using Droplets Generated and Measured in Continuous Flow Devices^a^^,^^b^

a Low concentrations/linear range of Michaelis–Menten plot not captured. n.a.: not applicable.

b Table updated from ref (306).

**Table 9:**
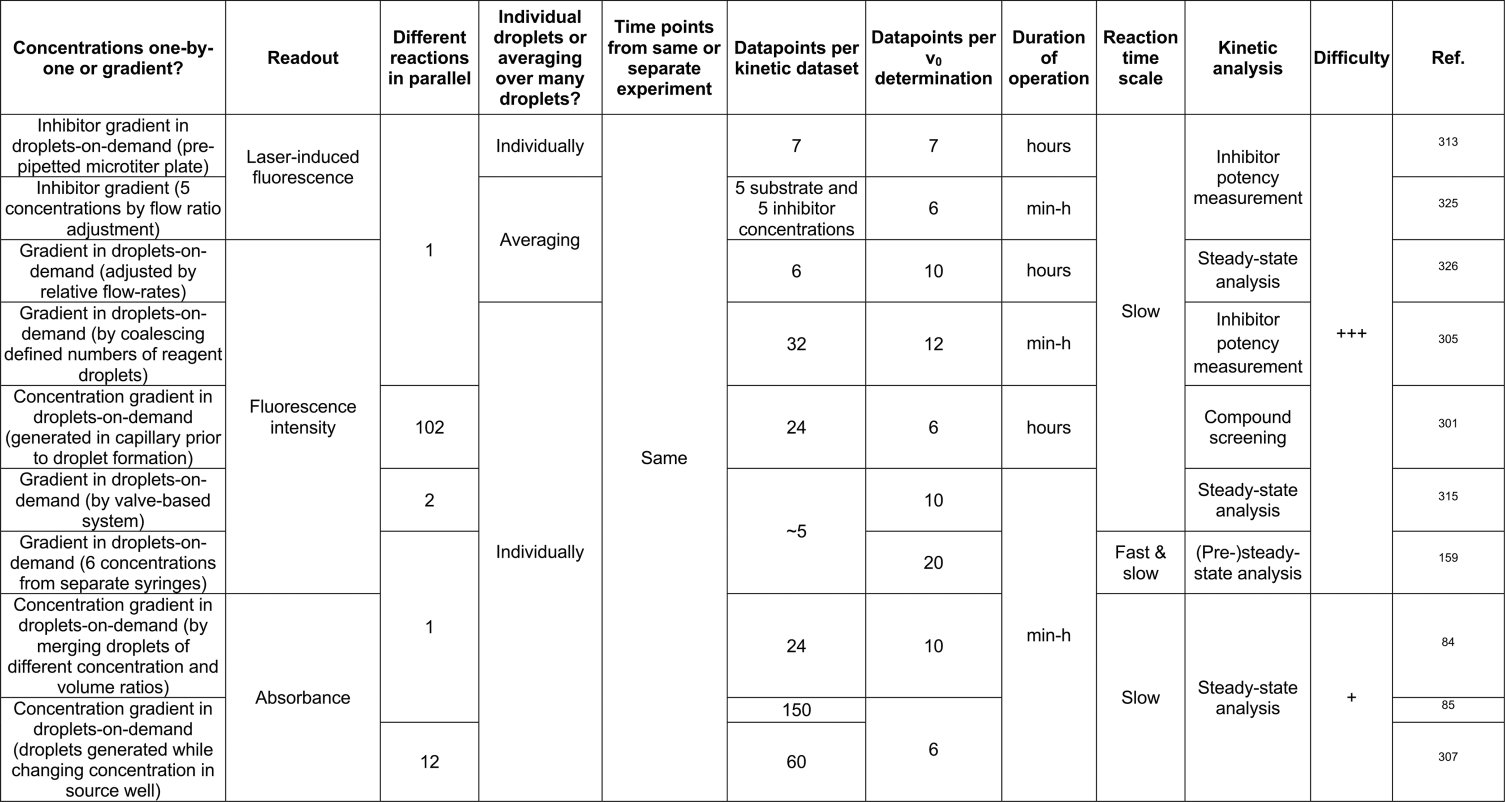
Microfluidic Systems for Kinetic Analysis Using Droplets in Segmented Flow^a^

a Table updated from ref (306).

**Table 10:**
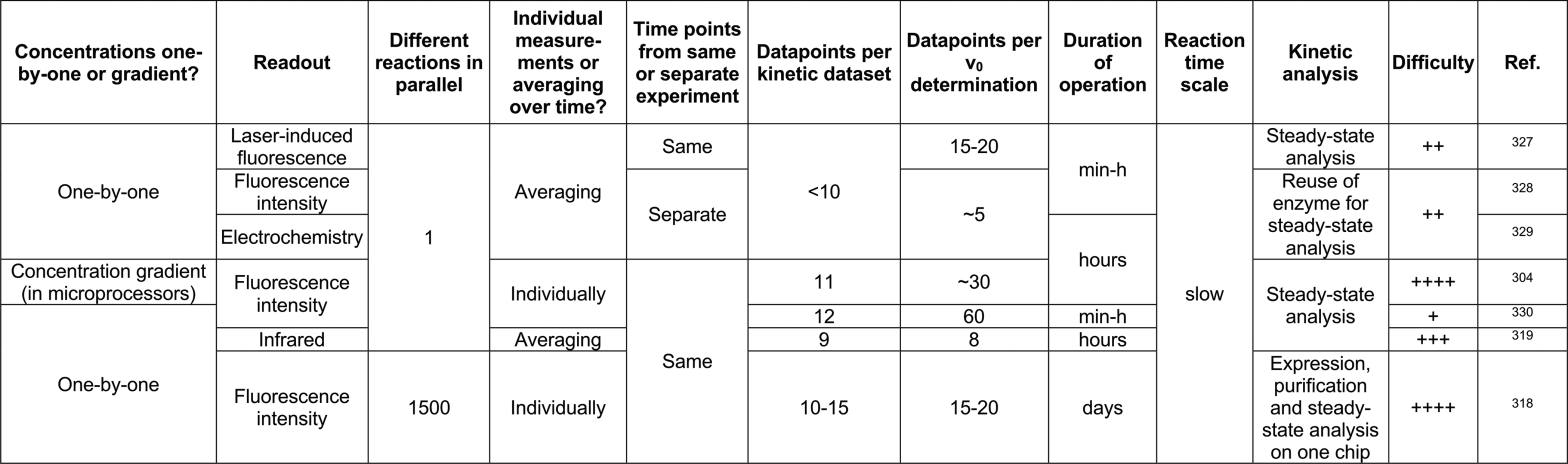
Droplet-Free Microfluidic Systems for Kinetic Analysis^a^

a Table updated from ref (306).

When they involve segmented flow (i.e., droplets or plugs), the systems can be classified into two categories:

### 10.1 Droplet-on-Demand (DoD) Systems

Full control over the sequence and composition of each droplet yields rich data sets: every droplet provides information. Here, the confidence in the data obtained from each droplet is the crucial basis for reducing droplet numbers (in turn enabling lower reagent consumption) without a loss in information quality. Early DoD systems were too limited in throughput to be useful when, e.g., one Michaelis–Menten curve ideally requires tens of data points along a concentration gradient and many mutants need to be characterized. On-chip DoD platforms based on valves^307−310^ or high-precision dosing pumps that allow formation of droplets at the junction of multiple inlet ports^311^ have been used to generate larger (μL) droplets with highly accurate reagent dispensation to generate concentration gradients of analytes. Other systems require expensive robotics^254,312^ or sophisticated multilayer microfluidic chips with valves that require expertise in fabrication and operation.^303,304,313−315^

**Figure 17:**
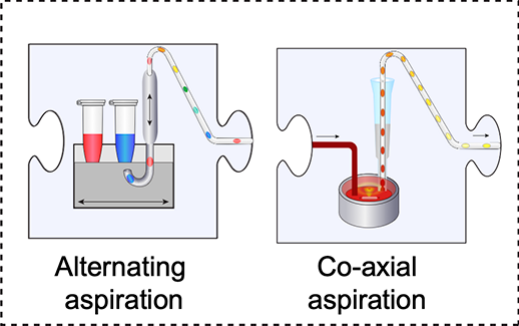
Droplet-on-demand systems. The content of each droplet in a sequence can vary by aspirating individual droplets from distinct aqueous solutions: either by alternately aspirating aqueous and oil phases or by aspirating from a well into an oil flow.

Technologically simpler alternatives have been developed (Figure 17): individual control over the size and content of droplets can be achieved with negative pressure that aspirates droplets, drawing defined volumes from reagent reservoirs, so that sequences of droplets with a dilution gradient emerge.^82,84,316^ Even simpler, coaxial aspiration from microwells can produce sets of droplets that reflect in their sequence the concentrations of reagents in the source well that are altered by injections during droplet formation. In 5 min, 150 combinations of reaction components (enzyme/substrate/inhibitor) can be produced and measured,^83^ and multiplexing can further increase the throughput.^306^ Such DoD systems can automatically create substrate concentration gradients and are suitable for deriving Michaelis–Menten parameters.^83,306^

A completely different approach was taken by Miller et al.,^301^ who generated a concentration gradient by Taylor–Aris dispersion and segmented the gradient microfluidically into droplets (140 pL). Here, the low confidence in the data obtained from single droplets required 10,000 data points to be measured in order to determine an IC_50_ value by massive statistical averaging.

A taste of the information obtained by completely miniaturized enzyme screening is given by Markin et al.,^317^ who developed the most comprehensive analysis tool to date, albeit in chambers rather than droplets. HT-MEK (high-throughput microfluidic enzyme kinetics) gave insight into stability and folding, enzymatic activity, and inhibition characteristics for more than 1500 mutants with high precision and within a few weeks. Practically, the reliance on valves complicates operation, and furthermore, some conditions have to be met (a fusion protein must be *in vitro* expressed and a fluorogenic assay available) and may limit the convenience of its use,^317^ leaving room for more versatile systems even if they have a lower throughput. This is the type of data that DoD systems should be able to provide in the future.

### 10.2 High-Throughput Production of Droplets with Identical Composition

Instead of setting up every droplet with a unique combination of reagents or conditions, several existing microfluidic systems rely on the high droplet production frequency to rapidly produce droplets with identical contents that can be interrogated. The reaction conditions can also be incrementally adjusted e.g. by varying flow rates and equilibration (see Figure 4C). The data quality in such systems is high due to the averaging of measurements from many droplets with the same contents e.g. at various positions in a delay line.^288,293,304,313,315,318,319^ However, their throughput is limited, because these systems have to be reset, cleaned, and equilibrated for each new enzyme or variant, and the reagent consumption is multiplied compared to DoD systems, because identical droplets need to be produced. While some systems can reveal additional detail, e.g. very rapid, pre-steady-state kinetics,^156,293,313,320^ it is necessary to assess on a case-by-case basis whether a droplet-based system is providing an advantage in terms of reagent volumes used (over the duration of the entire experiment, not just per droplet), mechanistic insight, throughput, and ease of operation.

## 11 Perspectives: More of the Same (Albeit Faster) Or Entirely New Ways of Working?

**Figure 18:**
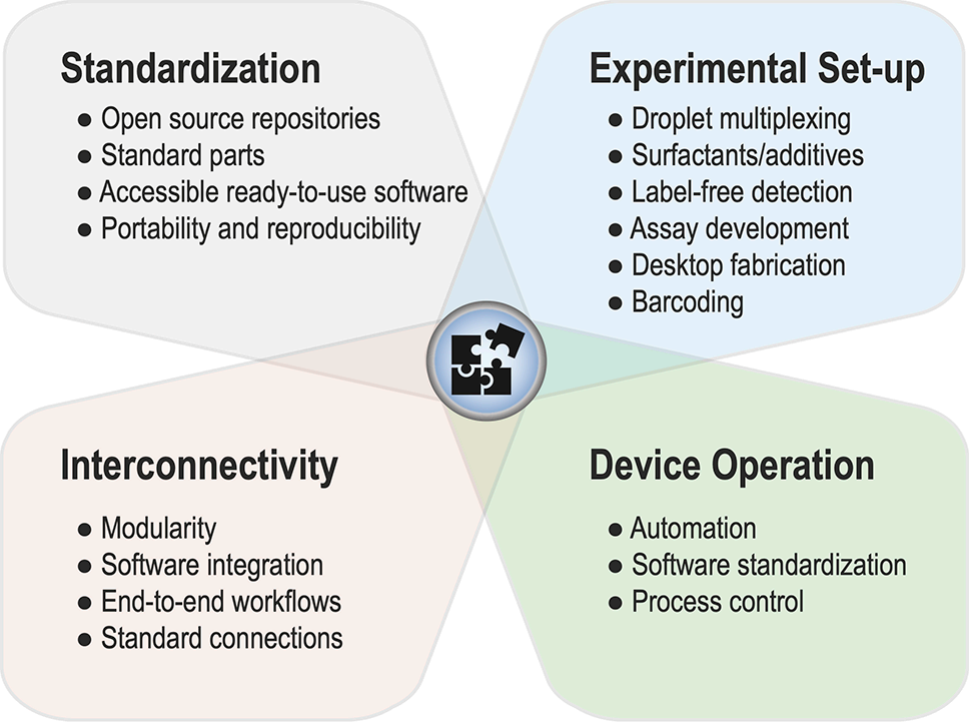
Areas for innovation. Droplet microfluidics, while a proven technology for protein engineering and single-cell analysis, still has areas for innovation. *Standardization*: Standardization of parts, designs, and software will allow greater portability and reproducibility of microfluidic experiments between research groups. This can be supplemented with designated online open-source repositories to enable rapid sharing of designs worldwide. *Experimental setup*: a host of areas for improvements in the experimental setup will allow the experimenter to access new ways of performing manipulations of droplets and open up new reaction types. Additionally more rapid prototyping methods are needed to iterate on designs during the experimental process. *Interconnectivity*: a great challenge for droplet microfluidics is to overcome issues when adding unit operations together, a large amount of the problem having to do with pressure differences in the device and the need for end-to-end workflows. Solving this problem will therefore lead to more complex devices becoming feasible. Integration of software and standard connections will reduce the incompatibility between set-ups. *Device operation:* automation of all on-chip processes, including droplet tracking and real-time feedback, will lead to the ability for process control of microfluidic devices. This requires software standardization which will increase accessibility of droplet microfluidics.

Despite the emerging track record of droplet microfluidics, several issues remain that prevent it from becoming the *de facto* standard for high-throughput experiments (Figure 18).

### 11.1 Accessible Microfluidic Devices for the Future

A critical issue is a lack of standardization in the community, leading to siloed designs and “reinventing the wheel”, amounting to wasted efforts and resources and a high entry barrier. Looking toward engineering disciplines, standardization of parts and open-source repositories are key in allowing rapid iterative improvements on designs. Analogous to programming, the ability to rapidly build up on others’ designs leverages the power of the community toward synergistic improvement. Commercialization of microfluidics has seen the introduction of standard designs, for example, the Luer lock and standard droplet-making chips. However, portability and reproducibility of experiments have room for improvement before a greater research community can readily adapt them, and the lack of a baseline microfluidic template prevents design iteration between groups.

The difficulty in simulating microfluidic devices, both continuous and droplet microfluidics, is due to the difficulty in solving the Navier–Stokes equations for complex geometries. As such, the computational demand makes this a challenging and costly endeavor, meaning that most groups use a trial-and-error approach based on historical designs. Innovation in the production of desktop fabrication methods could lead to more rapid design cycles through trial and error. Several groups have worked on creating software for the generation of microfluidic devices, e.g. a suite of software for design automatization.^330^ This DAFD platform is a web-based application that can predict the performance of microfluidic devices and automate the design.^330^ Taking inspiration from the electronics industry, MINT, a hardware language for describing components and devices for microfluidic devices,^331^ was developed.

### 11.2 Tracking the Identity of Samples

Compared to the microtiter plate, tracking the identity of a particular sample in droplet microfluidics is nontrivial, since millions of droplets are typically involved in any one droplet microfluidic experiment. Droplets normally travel in single file, and so the droplet’s chronological position can serve as an ID. However, it is difficult to maintain this sequential pattern, since the downstream analysis of the droplet contents destroys this position (droplets are usually collected in bulk after microfluidic analysis, and therefore, the positional information is lost). Furthermore, even if a droplet can be tracked, there are several problems in identifying a particular droplet, since their contents are not easily decipherable to an assay readout. This problem has been tackled in two different ways: optical and genetic encoding, which are both methods of barcoding droplets.^332^ Optical encoding usually involves the addition of chromophores or fluorescent molecules to droplets. Diversity of the barcodes can be introduced through variation in concentration during droplet generation or through mixing particles with contents prior to encapsulation. For example, the diversity of a million optical barcodes has been shown through stochastic encapsulation of beads of slightly different diameters.^333^ Droplets can also be indexed to an array similarly to microtiter plates. For example, Cole et al. used a method of sorting for positive droplet hits and then dispensing them in an array fashion.^334^ Genetic barcoding has revolutionized the field of single-cell sequencing; the general strategy for these methods involves generating a library of barcodes using DNA oligonucleotides. Cell genetic contents can then be linked to a particular barcode, and only that droplet’s genetic contents will be associated. The limitation of this is that downstream sequencing is required to understand the contents of the droplets. There, therefore, remains a need for a high-throughput method to link the genetic contents of the droplet with the readout of the droplet, particularly for protein engineering, where the variant and phenotype need to be connected. For example, Abseq is a method for detecting epitopes of interest by linking antibodies with sequence tags allowing for multiplexing of protein expression in single cells.^335^ Increasing the number of “bins” can pool variants with similar characteristics together; for example, pooling different phenotypes into several bins using multiple sorting lanes has been shown.^203^

### 11.3 Complex Modular Devices

A challenge is building modular workflows on droplet microfluidics from several unit operations (e.g., droplet formation, picoinjection, sorting, and splitting) that mimic the macro-scale. However, problems may arise when trying to chain any individual operations. Typically, a microfluidic workflow with multiple steps is performed through multiple off-chip incubation steps using droplet chambers and re-injection steps. However, the chance of droplet instability increases with the amount of manual manipulation. Additionally, the complexity of droplet routing increases as the design complexity increases. It becomes very difficult to predict the flow behavior of droplets, leading to many device iterations to get this correct. Additionally, due to the unpredictability of flow, minor design changes can lead to unwanted effects and therefore need to be empirically tested and subjected to iterative improvements to obtain the correct design. Even a brief incubation for an additional 5 min (on the macro-scale) can become a complex problem when adjusting a multi-operational microfluidic device. Increased device length leads to increased back-pressure: as the length of the device increases and the complexity of interconnected channels increases, this leads to regions of high pressure that are hard to simulate. Currently, software is ad hoc, and running the device requires the use of several programs (e.g., camera control, real-time analysis, and pump operation). Integration of software would allow for more streamlined experiments and true digitization of the experiment carried out by having a digital record of all parameters. Furthermore, different microfluidic modules often require different droplet frequencies; for example, droplet generation can be performed at tens of thousands of hertz, whereas sorting generally occurs at hundreds or thousands of hertz. Trying to balance modules that have different operations and frequencies therefore requires attention. Examples of strategies to create modular microfluidics^336−340^ have used a general strategy to link together microfluidic “blocks” through Luer connectors or smooth seals. More insight is required in understanding flow properties in more complex integrated chips and designing truly end-to-end chips.

### 11.4 Device Operation—Will There Ever Be One Device for All Directed Evolution Experiments?

We have visualized the design of droplet microfluidic workflows as connecting jigsaw pieces,^341^ but questions of efficient integration remain: with increased device length comes increased pressure and complexity of interconnected channels, the consequences of which are hard to simulate. Different microfluidic modules often require different droplet frequencies (e.g., for droplet generation, which is often >10 kHz, compared to subsequent sorting which is often well below this value). Due to the large number of physical variables present when conducting a microfluidic experiment, even slight variations can lead to various problems. Computer vision can provide a potential solution to these problems, both in the setup and running of the microfluidic device. For example, by linking visual cues to the automation of pumps, variability or anomalous events can be countered by identifying the problem. Additionally, it may be helpful for devices to have a “flushing” regime in which the experiment can be automatically halted, flushed into a waste outlet, with subsequent reconfiguration of the setup. Valves and computer vision provide a possible way of realizing such a design improvement. Automation of microfluidic devices is a promising route for microfluidics to achieve the same widespread use. A large part of the lack of implementation of droplet microfluidics as a standard for protein engineering likely lies within the difficulty in setting up and running the device and inertial adoption issues. A device setup whereby fluidic control, pressure issues, troubleshooting, droplet tracking, and analysis are contained and automated within the microfluidic system (process control) would remove a large barrier to entry for many would-be end users.

### 11.5 Future Device Architectures

3D architecture from 3D printing opens the possibility for much more complicated and integrated microfluidic chips. The ability to design in 3D, as opposed to the traditional 2D or 2.5D used in conventional microfluidic designs, offers several advantages. For example, channels can cross each other without interference, electronics can be more easily integrated within the chip, and standard connections for chip-to-world and other microfluidic devices can be built into the device itself. A further advantage of 3D printing is that ideas can be easily shared and distributed among scientific laboratories, whereas the quality of soft lithography can be highly operator-dependent. Integrating electronics with microfluidics is another avenue by which microfluidic functionality and ease of use can be expanded upon, the benefit being the portability of microfluidic devices with embedded electronics.

### 11.6 Key Technology Benefits of Droplet Microfluidics

Droplet microfluidics offers several key benefits that make it uniquely positioned to tackle biochemical problems, above and beyond other methods of enquiry.

#### 11.6.1 Savings

Combinatorial approaches such as directed evolution and functional metagenomics are becoming increasingly popular, but their scale comes at a price. Liquid handling robots automatize steps that are normally carried out by manual pipetting and reach throughputs on the order of 10,000 per week.^342^ Plasticware and consumables have to be factored in as running costs as well as reagent consumption. Droplet-based approaches achieve massive miniaturization: in assay volume (10^6^-fold from pL to μL), in plasticware (an afternoon’s droplet experiment with ∼10^7^ assays would require more than 26,000 384-well plates), and in total reagent volume (from thousands of liters to tens of μL). Agresti et al.^122^ calculated a million-fold decrease in cost (based on capital expenditure of several millions, plus staff).

In droplet microfluidics hard- and plasticware are largely replaced conceptually by a separation between phases, manipulation (routing and processing of droplets through active or passive methods), and *in situ* analysis of components. The maximum speed (and the throughput per time) is currently 1000-fold greater than robotics.^122^ Additional factors such as evaporation and capillary action limit the maximum throughput of any robotic microtiter plate screening assay, since liquids will tend to “stick” to pipet ends or rapidly diffuse into the surrounding environment. On the other hand, section 9 outlines experimental challenges that are in turn intrinsic to work in droplets: overcoming leaking requires new substrates, oils, and surfactants and may need to be adjusted for every new reaction. Droplet stability is important for maintaining the monoclonality and impermeability of droplet compartments and, especially, in multistep workflows. The prerequisite for high fidelity of manipulation steps is based on the fluid dynamics of uniformly sized, structurally stable droplets.

#### 11.6.2 Combining High-Throughput Selections with High-Throughput Analysis

The logic “*more at lower cost is better*” is compellingly universal when applied to screening, but there are approaches that realistically can *only* be addressed when an ultrahigh-throughput system is available:

##### 11.6.2.1 Functional Metagenomics

The search for rare “needles” in the “haystack” of metagenomic DNA is an example that will benefit enormously from faster exploration by droplet approaches. The environment provides a rich recourse of enzymes with activities that can be harnessed in industrial biocatalysis. Yet, hits are very rare (estimated to be 1 in 10^3^ to 10^5^ library members or less, depending on the prevalence of the starting activity in the source microbiome).^343,344^ Droplet campaigns from million-membered libraries ended up with just a handful of hits,^118,131,196^ emphasizing that success was only possible with a throughput on the order of millions, while a throughput of around 10,000 (as in robots^342^ or colony screening^345^) would have gone nowhere.

We envisage a broader role of droplet microfluidics in exploring the functional repertoire of the natural environment, to build up and expand our repertoire of biocatalysts. Such enzymes can be presumed to exist in the biosphere, but an overwhelming majority of them have not been discovered. The sequencing of environmental DNA is now fast and cheap so that metagenomic databases are growing exponentially (e.g., EMBL’s MGnify database now has more than 2.3 billion open reading frames),^346^ but minuscule reliable functional knowledge is recorded. Indeed, their automatic assignment to a putative function is somewhat deceptive: very few activities of open reading frames in these databases have been experimentally verified (compared to the large number of open reading frames that have never been studied in wet lab experiments). When simple sequence comparisons are used, predictions of very closely related activities may be reliable. But deriving new functions (or even promiscuous side activities that are useful starting points for evolution) from sequence comparisons is limited because we have not annotated enough sequences functionally based on experimental evidence to allow confident prediction. It remains to be seen when a sufficient number of reliable assignments is available to understand the functional potential of all deposited sequences.^196^

The advent of AlphaFold2^347^ may make reliable structures available without the need to express and crystallize proteins. Nevertheless, the prediction of the function of these structures is difficult or impossible, even if the structural model is close to reality. The key problem of relating sequence (or structure) with function is unresolved. Unearthing valuable functional information on metagenomes in rapid (>kHz) and resource-saving (pL) fashion in droplet-based approaches will facilitate capturing information to bring about a comprehensive understanding of the determinants of function (and where in “sequence space” they are found). This functional metagenomics approach will be the basis for correct annotation, which in turn allows classification (and reclassification) to improve currently imperfect databases (such as CAZy^348,349^). Promiscuous activities are highly interesting as a springboard for the evolution of new function^194^ but are unpredictable, necessitating experimental evidence. The interplay of experimental functional annotation obtained at ultrahigh-throughput, bioinformatics, *in silico* modeling, and harvesting of database information will be a powerful combination in enzyme discovery, no doubt in the future aided by machine learning and other artificial intelligence methods.

##### 11.6.2.2 Mapping Fitness Landscapes: From Epistasis to Predictive Biology?

The idea of walking through fitness landscapes has been used as a metaphor for the process of evolution. The shape of these landscapes is currently unpredictable; so, the more of this sequence space we can explore empirically, the greater the chances of finding hits and of empirically understanding how to navigate it (to obtain a notion of how evolvable an enzyme is).

**Figure 19:**
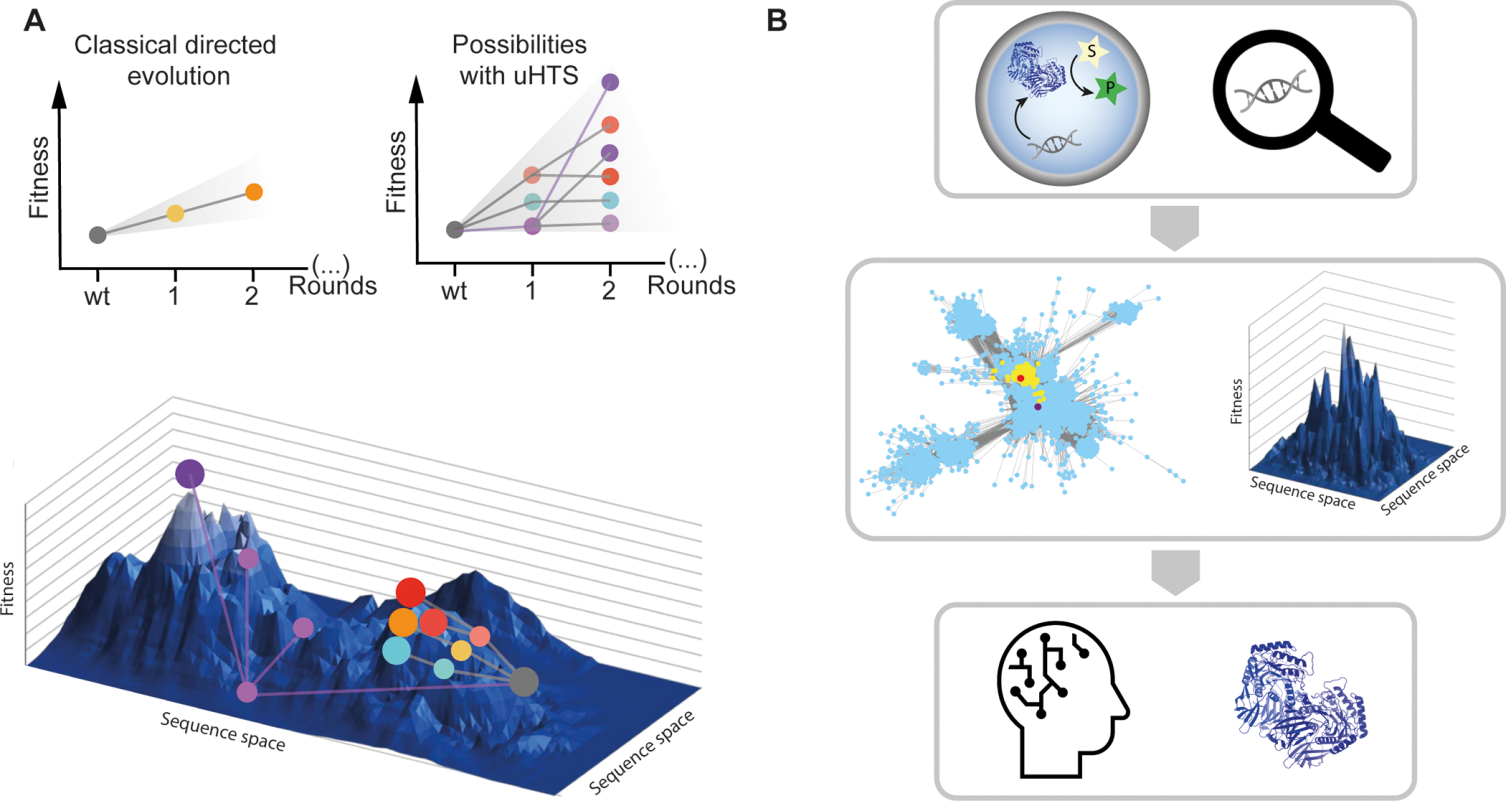
Perspectives. The impact of ultrahigh-throughput screening on directed evolution. (A) Classical directed evolution constrains the campaign to the most improving variants after each round. This can yield highly improved variants in a very economical fashion but restricts the exploration of sequence space to one trajectory. With uHTS, multiple trajectories can be explored in an unbiased manner, also allowing rounds with less stringent screening regimes, increasing the likelihood of encountering synergistic effects or one-in-a-million events. (B) Droplet-based ultrahigh-throughput screening and characterization allows functional annotation of sequence space (left). Sequence similarity network from Neun et al.^196^ showing a novel bridgehead for functional annotation of GH3 β-glucoronidases (red). An already annotated/characterized GH3 β-glucoronidase is shown in purple while sequences directly connected to the novel bridgehead are shown in yellow. Blue sequences show all significant search hits from a MGnify query. Using ultrahigh-throughput screening coupled to high-throughput sequencing, the effect of mutations on an enzyme can be characterized on a large scale (right). Combined, we envision this large-scale sequence–function mapping to provide data for the next generation of AI-based enzyme discovery and engineering efforts.

(i) *Synergistic interactions*. The possible combinatorial diversity of mutations is vast. However, randomizing single positions ignoring combinatorial effects of mutations often misses out on potential improvements by synergy.^350,351^ Moreover, epistasis-induced path dependence of directed evolution can limit the number of available productive trajectories. Consequently, trajectories to higher fitness are rare,^351−353^ so that ultrahigh-throughput screening is necessary to identify productive trajectories based on synergistic combinations of mutations against the odds.
(ii) *Focused vs unbiased exploration of sequence space*. Sequence space is vast and can never be screened in its entirety (e.g., a 100 amino acid-long sequence can encode 20^100^ different proteins). Ultrahigh throughput provides the means for screening focused libraries of four to five completely randomized positions.^123,128^ Recent examples have shown that this enormous throughput can be used to improve biocatalysts 960-fold with extensive remodelling of the active site with only one round of directed evolution.^123^ Another focused library also led to rapid evolutionary improvements of a phosphotriesterase.^117^ Indeed, such “smart” libraries^354,355^ are often used to increase the chance of success of directed evolution campaigns. This has helped, especially when only low-throughput screens were available, but the library design also limits the outcomes eventually. Wrenbeck et al.^356^ have shown that substrate specificity is globally encoded. In addition, the largest improvements observed in the directed evolution of enzymes^357,358^ were not obtained from smart libraries. This raises the question of whether the unbiased exploration of sequence space using error-prone PCR libraries is sufficient if an ultrahigh-throughput screen is used. Ultrahigh-throughput screening can be used to escape stalled evolutionary trajectories by providing access to large leaps in sequence space^128^ or by enabling the introduction of mutations that bypass negative epistasis.^353^ The strength of droplet microfluidics would be to carry out many screens at a low cost and proceed through multiple rounds, even without characterization. This practice would be a break with the way how directed evolution has largely been carried out thus far. Historically many directed evolution campaigns remained highly bottlenecked, as after each round the best variant was chosen and used as a template for further library design.^358^ Such a strategy is the most economical one when only a low-throughout screening system is available: there is simply no capacity to carry forth multiple starting points. However, the focus on one (or a few) “best” mutant(s) misses out on permissive mutations that allow the fixation of highly improving further mutations.^352,359^ The availability of ultrahigh-throughput screening makes it possible to tolerate additional phylogenies that are not “best” in each round. They can be carried on into subsequent rounds in an inclusive fashion, where they may develop and “overtake” the frontrunners in earlier rounds (Figure 19A).^35^ It remains to be seen whether this practice of simultaneously entertaining *multiple* trajectories in one experiment will overcome the “diminishing returns” syndrome^358^ that describes a situation in which long-term evolution comes to a halt after several rounds in a quasi cul-de sac. The ultrahigh droplet screening capacity thus changes the experimental options for exploring strategic options in directed evolution, affording the combinatorial luxury of relaxed stringency. The change of strategy (from “bottlenecked” to “inclusive”) ties in with playing out alternative evolutionary scenarios, including a neutral drift regime that carries over a set of the best variants from each round.^360−364^ Neutral drift was applied in the evolution of an arylsulfatase (ultimately resulting in a >100,000-fold improvement) with medium-throughput screening (of ∼10,000 colonies), which remained unsuccessful prior to a “blind” neutral drift.^357^ Characterizing the mutant networks (e.g., by analysis of the kinetics and structures of each) would reveal the roles that individual residues and their combinations play, but performing extensive analysis for the outcomes of each round will stretch the capacity of most laboratories (even when the methods outlined in section 10 are used). Instead, one could go through multiple rounds of droplet screening and adjust the selection threshold to enter phases of adaptive vs nonadaptive (tolerant) regimes while always recovering not just a few but many mutants (so that entertaining multiple trajectories becomes plausible). Progress in microfluidic design and detection technologies (as outlined above) makes it less of a leap of faith to sort “blindly” without round-by-round characterization, but instead with reliable control of a selection threshold set by the operator to ensure a sufficient number of clones is recovered to capture multiple trajectories. Like continuous evolution,^365,366^ such an approach would traverse large areas of sequence space quickly by virtue of the ultrahigh-throughput possible in droplet microfluidics. Not only would trajectories be explored, but *multiple* trajectories can be recorded (by next-generation sequencing) and characterized ex post, when frontrunners have been chosen by investigating their origin in a sequence network. It remains to be seen whether long-term—blind but traceable—evolution will generate data sets that not only record the history of an emerging functional protein but are also able to predict where future improved mutants can be found without additional experiments.
(iii) *Sequence description of evolutionary trajectories as unique data sets for AI/ML analysis*. The combination of droplet-based ultrahigh-throughput screening (UHTS) and next-generation sequencing (NGS) (deep mutational scanning; DMS) gives access to large-scale sequence–function maps of enzymes (fitness landscapes).^367^ Droplet-based deep mutational scanning allows the deciphering of the encoding of fundamental enzyme properties such as enzymatic activity, thermodynamic stability, and substrate specificity^111,135,368^ which will be facilitated by the adaptation of novel workflows to disentangle enzyme expression level and activity.^27,369^ Novel long-read-based methodologies such as Oxford Nanopore^370^ and PacBio^371^ sequencing facilitate the resolution of epistasis in evolutionary trajectories^125^ which previously relied on complex workflows combining short reads with an upper limit for gene length.^372^ Nevertheless, very few such extensive data sets exist. DMS data have been used not only to infer information on single enzymes but also to extrapolate from it by machine learning,^373^ resulting in novel binders^374,375^ and industrially relevant biocatalysts.^376^ Intriguingly, machine learning can also be used to extrapolate into previously unexplored territories of sequence space, generating functional enzyme sequences solely based on observed sequence diversity.^377^ We envision that increased availability of data on the encoding of function by exploration of sequence space using DMS and functional metagenomics combined with more efficient machine learning algorithms^378^ will inform *in silico* directed evolution with higher fidelity (Figure 19B). In this scenario, droplet-based UHTS would not only elicit new functional proteins but also provide the data necessary for the *insilico* generation of the next wave of protein binders and biocatalysts.

## 12 Conclusions

In little more than two decades, ultrahigh-throughput assays in droplet compartments have come a long way from proof-of-principle enrichment experiments to identifying novel functional proteins for a range of target reactions in microfluidic devices screening almost routinely with high analytical precision on a scale of more than a million library members per day. The field is now poised to take advantage of the potential for automation at low capital expenditure and a step change in speed and capacity, while avoiding plasticware waste. The open source availability of device designs and the prospect of modular workflows and of interfacing custom-made devices with established flow cytometry facilities will lower the access barrier for new users. Fast design/testing cycles enabled by, e.g., soft lithography or in the future by benchtop 3D printing will put microfluidic devices rapidly in the hands of users. The chemical versatility of droplet screening is boosted by the increasing coverage of different chemical transformations and enzyme classes (directly and through coupled assays). An emerging framework for troubleshooting and protocol adjustments ensures that tailor-made assays can be implemented. Taken together, these advances will equip a broader circle of practitioners to use droplet microfluidics and establish accelerated protein engineering campaigns in the toolkit of the protein engineer.

Protein engineering has been rapidly revolutionized by the integration of next-generation sequencing, AI/ML-enabled structural modeling,^347^ and integration with comprehensive databases (e.g., the MGnify database containing >2 billion open reading frames^346^). All these approaches are, however, unable to reliably predict function: functional assignments still have to be experimentally addressed, making this the rate-limiting step in discovery efforts. Droplet microfluidics will accelerate the slowest process in protein engineering, and its increases in throughput and speed will resonate well beyond the increased convenience of faster and cheaper screening. Tracking the dynamics of evolution not only in genotype-space but also at the level of phenotype (e.g., catalysis or binding) will generate data sets that will overcome current “blind” discovery campaigns and “map” navigation through the vastness of sequence space in the search for novel functional proteins.
